# Single-molecule analysis reveals rotational substeps and chemo-mechanical coupling scheme of *Enterococcus hirae* V_1_-ATPase

**DOI:** 10.1074/jbc.RA119.008947

**Published:** 2019-09-13

**Authors:** Tatsuya Iida, Yoshihiro Minagawa, Hiroshi Ueno, Fumihiro Kawai, Takeshi Murata, Ryota Iino

**Affiliations:** ‡ Institute for Molecular Science, National Institutes of Natural Sciences, 5-1 Higashiyama, Myodaiji-cho, Okazaki, Aichi 444-8787, Japan; § Department of Functional Molecular Science, School of Physical Sciences, SOKENDAI (Graduate University for Advanced Studies), Shonan Village, Hayama, Kanagawa 240-0193, Japan; ¶ Department of Applied Chemistry, Graduate School of Engineering, University of Tokyo, 7-3-1 Hongo, Bunkyo-ku, Tokyo 113-8656, Japan; ‖ Department of Chemistry, Graduate School of Science, Chiba University, 1-33 Yayoi-cho, Inage-ku, Chiba 263-8522, Japan; ** Japan Science and Technology Agency (JST), PRESTO, 1-33 Yayoi-cho, Inage-ku, Chiba 263-8522, Japan

**Keywords:** ATPase, biophysics, Enterococcus, molecular motor, single-molecule biophysics, vacuolar ATPase, ion pump, rotary motor, rotational substep, V1-ATPase

## Abstract

V_1_-ATPase (V_1_), the catalytic domain of an ion-pumping V-ATPase, is a molecular motor that converts ATP hydrolysis–derived chemical energy into rotation. Here, using a gold nanoparticle probe, we directly observed rotation of V_1_ from the pathogen *Enterococcus hirae* (EhV_1_). We found that 120° steps in each ATP hydrolysis event are divided into 40 and 80° substeps. In the main pause before the 40° substep and at low ATP concentration ([ATP]), the time constant was inversely proportional to [ATP], indicating that ATP binds during the main pause with a rate constant of 1.0 × 10^7^ m^−1^ s^−1^. At high [ATP], we observed two [ATP]-independent time constants (0.5 and 0.7 ms). One of two time constants was prolonged (144 ms) in a rotation driven by slowly hydrolyzable ATPγS, indicating that ATP is cleaved during the main pause. In another subpause before the 80° substep, we noted an [ATP]-independent time constant (2.5 ms). Furthermore, in an ATP-driven rotation of an arginine-finger mutant in the presence of ADP, −80 and −40° backward steps were observed. The time constants of the pauses before −80° backward and +40° recovery steps were inversely proportional to [ADP] and [ATP], respectively, indicating that ADP- and ATP-binding events trigger these steps. Assuming that backward steps are reverse reactions, we conclude that 40 and 80° substeps are triggered by ATP binding and ADP release, respectively, and that the remaining time constant in the main pause represents phosphate release. We propose a chemo-mechanical coupling scheme of EhV_1_, including substeps largely different from those of F_1_-ATPases.

## Introduction

Vacuolar ATPase (V-ATPase)^3^ is a rotary molecular motor that actively transports ions across the cell membrane coupled with ATP hydrolysis (1). Eukaryotic V-ATPase functions as a proton pump that plays an important role in acidification of intracellular vesicles and is responsible for various cellular processes, such as pH homeostasis, membrane trafficking, endocytosis, and protein degradation (2–4). A bacterial V-ATPase from *Enterococcus hirae* (EhV-ATPase) functions as an ion pump driven by ATP hydrolysis similar to eukaryotic V-ATPase and actively transports sodium ion instead of proton (5). On the other hand, some bacterial V-ATPases, such as *Thermus thermophilus* V-ATPase (6), act as ATP synthase in the cell, similar to A-type ATP synthase, which is found in archaea (7). Therefore, bacterial V-ATPase is also called V/A-ATPase. In this study, we describe EhV-ATPase as V-ATPase rather than V/A-ATPase to clarify the difference in physiological function.

The V-ATPase is composed of two rotary motors, a membrane-embedded V_o_ transporting ions and a water-soluble V_1_-ATPase (V_1_) hydrolyzing ATP, which are connected by a central stalk and either two (EhV-ATPase and V/A-ATPase) or three (eukaryotic V-ATPase) peripheral stalks (8–13). The V_o_ is composed of the channel-forming *a* subunit, the ion binding ring of the *c* subunits, and the *d* subunit and the EG subcomplex, which form the central and peripheral stalks, respectively (14, 15). The V_1_ is composed of A, B, D, and F subunits, in which three AB pairs form a hexagonally arranged stator A_3_B_3_ ring, and the DF subcomplex is a rotor inserted into the A_3_B_3_ ring (16–18). The catalytic sites are located at the interfaces of the A and B subunits, and when the ATP molecules are hydrolyzed sequentially at the three catalytic sites, the DF rotates unidirectionally in the counterclockwise direction viewed from the V_o_ (membrane) side. However, the chemo-mechanical coupling mechanism of V_1_ has not been fully understood (19–21).

Another well-studied ATP-driven rotary molecular motor is F_1_-ATPase (F_1_), a water-soluble portion of F-type ATP synthase. The chemo-mechanical coupling mechanism of F_1_ has been investigated in detail by single-molecule analysis using F_1_ from thermophilic *Bacillus* PS3 (TF_1_) (22–28) and F_1_ from human mitochondria (HF_1_) (29). TF_1_ and HF_1_ rotate counterclockwise with 120° steps per ATP hydrolysis. The 120° steps of TF_1_ and HF_1_ are further divided into 80 and 40° substeps and 65, 25, and 30° substeps, respectively. The first substeps (80° of TF_1_ and 65° of HF_1_) are triggered by ATP binding coupled with concomitant ADP release from another catalytic site. The second substeps (40° of TF_1_ and 25° of HF_1_) are triggered by the release of P_i_. The third 30° substep of HF_1_ is triggered by ATP cleavage, whereas ATP cleavage induces only small rotation in TF_1_ (30). The single-molecule analysis has also been performed using F_1_ from *Escherichia coli* (EF_1_) (31–33) and yeast mitochondria (YF_1_) (34). It has been confirmed that the 120° steps of EF_1_ and YF_1_ are further divided into 85 and 35° substeps and 87 and 33° substeps, respectively. In EF_1_, it has also been shown that ADP is released at 50° after ATP binding during the first substep (35), but the detail of chemo-mechanical coupling mechanism is not as clear as TF_1_ and HF_1_.

For F_1_, not only information of dynamics obtained by single-molecule analysis, but also high-resolution structures that correspond to elementary steps of the chemo-mechanical coupling cycle, have been obtained using F_1_ from bovine mitochondria (BF_1_) (36–42), YF_1_ (43–46), EF_1_ (47, 48), and TF_1_ (49, 50). The chemo-mechanical coupling scheme of F_1_ has been substantially characterized from the aspects of the single-molecule dynamics and atomic level three-dimensional (3D) structural analyses.

In the study of V_1_-ATPase, the rotation has been directly visualized for the first time by single-molecule analysis using V_1_ (or V_1_/A_1_) from *T. thermophilus* (TtV_1_). As F_1_, the TtV_1_ rotates counterclockwise with 120° steps per ATP hydrolysis, but no substeps have been resolved (19, 20, 51). The high-resolution 3D structure of TtV_1_ has also been solved by X-ray crystallography (52, 53). Furthermore, by cryo-EM, 3D structures of intact *T. thermophilus* V/A-ATPase in three main rotational states, in which orientations of the rotor subunits are different by ∼120° from each other, have been solved (11, 12, 54). Interestingly, in the most recent high-resolution structures (54), two additional substates, in which the whole V_1_ portion is slightly twisted against the V_o_ portion to the ATP synthesis or hydrolysis direction by 8 or 10°, respectively, have also been identified. However, the detailed comparison between the single-molecule dynamics and the 3D structure has not been conducted yet, and a chemo-mechanical coupling scheme remains elusive.

As a bacterial V_1_, we have previously visualized the rotation of V_1_ from *E. hirae* (EhV_1_) (21). In the previous study, using a gold nanoparticle as a low-load probe, we have found two distinct reversible rotational states, clear and unclear (21, 55, 56). In the clear state, EhV_1_ showed counterclockwise and 120° stepwise rotation without a substep, which is similar to TtV_1_. In contrast, in the unclear state, the probe attached to rotor DF showed large fluctuation. The clear and unclear states were not only observed in EhV_1_ reconstituted from the isolated stator A_3_B_3_ ring and the rotor DF subcomplex, but also in the recombinant A_3_B_3_DF complex, which has AviTag (57) in the D subunit for probe attachment. From these results, we previously concluded that the unclear state is an intrinsic property of the isolated EhV_1_, caused by the unstable interaction between the stator A_3_B_3_ ring and the rotor DF subcomplex. Supporting this notion, the whole EhV-ATPase complex with two peripheral stalks did not show an unclear state (55). In the studies of EhV_1_, in addition to the single-molecule analysis of rotational dynamics, high-resolution structures with different conformations have been solved by X-ray crystallography (16, 17). Based on insights obtained so far, a model of a chemo-mechanical coupling scheme of EhV_1_ without substeps has been proposed (17, 56).

In our previous single-molecule study (21), the EhV_1_ contained additional amino acid residues added to the N terminus of the D subunit and the C terminus of the F subunit for protein purification or inserted in the middle of the D subunit for AviTag modification. In the present study, we found that these additional amino acid residues caused the unclear state. Newly constructed EhV_1_ with minimized additional amino acid residues showed only clear states and substeps in the rotation. We performed single-molecule analysis using this newly constructed WT EhV_1_ and arginine finger (Arg-finger, Arg-350 in the B subunit (58–60)) mutant EhV_1_ (A_3_B(R350K)_3_DF complex), which has very low ATPase activity (16). From the results of detailed single-molecule analyses and the previous structural information (16, 17), here we propose a new model of a chemo-mechanical coupling scheme of EhV_1_ including substeps.

## Results

### EhV_1_ shows clear rotation with substep

The new construct of EhV_1_ (Fig. S1*A*), which is different from those used in the previous study (21), was prepared, and ATP-driven rotation was observed. One of the previous constructs has an additional 7 amino acid residues (GSSGSSG) at the N terminus of the D subunit and 12 amino acid residues (SGPSSGENLYFQ) at the C terminus of the F subunit, after removal of tags for purification. Another previous construct has the AviTag, which consists of 15 amino acid residues (GLNDIFEAQKIEWHE) (57), inserted between Gly-121 and Tyr-122 of the D subunit for biotinylation. In the present study, we have prepared a new construct of the EhV_1_, which has a glycine residue instead of methionine at the N terminus of the D subunit and an additional 7 amino acid residues (GSSGSSG) at the N terminus of the F subunit. The newly constructed rotor DF subcomplex was purified and reconstituted with the stator A_3_B_3_ ring to form the EhV_1_ complex (Fig. S1*B*). In this study, this EhV_1_ complex is referred to as the WT.

**Figure 1.**
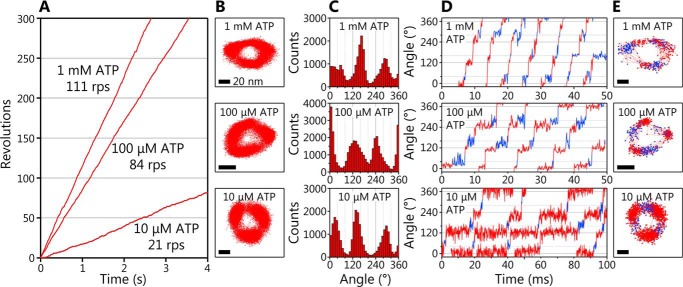
**Clear rotation and rotational substeps of EhV_1_.** *A*, typical time courses of rotation of WT EhV_1_ at 1 mm, 100 μm, and 10 μm ATP. The frame rate was 10,000 fps in all experiments. *B*, the *x-y* trajectories of the rotation shown in *A. Scale bar*, 20 nm. *C*, angle distributions of the rotation shown in *A. D*, expanded time courses of the rotary angle at 1 mm, 100 μm, and 10 μm ATP. *E*, *x-y* trajectories of the rotation shown in *D*. The main pauses and subpauses are shown in *red* and *blue*, respectively. *Scale bar*, 20 nm.

The rotation of the WT EhV_1_ driven by ATP hydrolysis was observed by single-molecule analysis probed with a 40-nm gold nanoparticle attached to the D subunit at 10,000 frames/s (fps). The rotating molecules were found at various ATP concentrations ([ATP]s) from 100 nm to 30 mm, whereas it was difficult to find rotating molecules at [ATP] lower than 10 μm in the previous study (Fig. S1*C*). The typical examples of the rotation at 10 μm, 100 μm, and 1 mm ATP are shown in Fig. 1. At any [ATP]s, the EhV_1_ showed only clear rotation (Fig. 1*A*), which is different from the previous study showing the clear and unclear rotations (21). The EhV_1_ showed stepwise rotations with pauses roughly separated 120° each other at each [ATP] (Fig. 1, *B* and *C*). Furthermore, when time courses of the rotational angle were carefully observed, we found short subpauses with the length of a few milliseconds between the main pauses separated by 120° (*blue lines* and *dots* in Fig. 1, *D* and *E*, respectively). This result indicates that the 120° steps are further divided into two substeps. The subpauses were observed at all tested [ATP]s. It seems that the duration of main pauses became shorter as [ATP] increased, whereas the duration of subpauses did not change.

### [ATP] dependence of rotation

**Figure 2.**
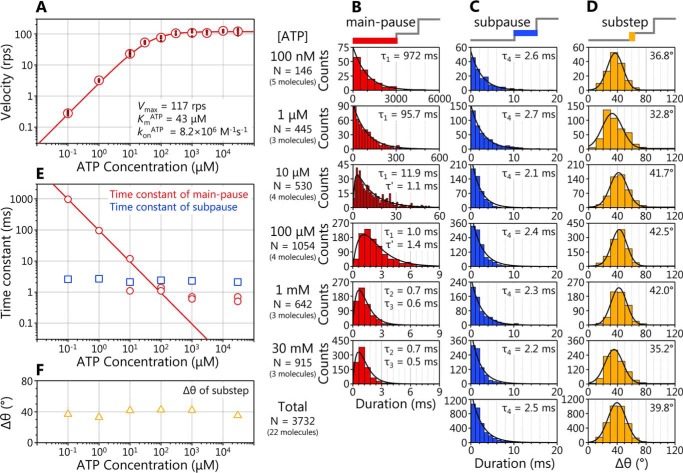
**Dwell time analysis of main pause and subpause.** *A*, [ATP] dependence of rotation velocity of WT EhV_1_. The *red open circles* and *small filled circles* show the average velocity and velocities of individual molecules, respectively. *Red line*, fit with the Michaelis–Menten equation: *V* = *V*_max_^ATP^ × [ATP]/(*K_m_*^ATP^ + [ATP]). The *V*_max_^ATP^ and *K_m_*^ATP^ were 117 ± 3 rps (fitted parameter ± fitting error) and 43 ± 6 μm, respectively. The *k*_on_^ATP^ was estimated from 3 × *V*_max_^ATP^/*K_m_*^ATP^ as 8.2 × 10^6^ m^−1^ s^−1^. *B*, distributions of duration of the main pause. For 100 nm and 1 μm ATP, the distributions were fitted with single-exponential decay functions: constant × exp(−*t*/τ). The time constants were obtained as 972 ± 41 and 95.7 ± 3.7 ms for 100 nm and 1 μm, respectively. From 10 μm to 30 mm ATP, the distributions were fitted with double-exponential decay functions assuming two consecutive first-order reactions: constant × (exp(−*t*/τ) − exp(−t/τ′)). The time constants were obtained as 11.9 ± 0.8 and 1.1 ± 0.2 ms for 10 μm, 1.4 ± 0.9 and 1.0 ± 0.9 ms for 100 μm, 0.7 ± 0.5 and 0.6 ± 0.5 ms for 1 mm, and 0.7 ± 0.5 and 0.5 ± 0.4 ms for 30 mm. *C*, distributions of duration of the subpause. The distributions were fitted with single-exponential decay functions: constant × exp(−*t*/τ). The time constants were obtained as 2.6 ± 0.2, 2.7 ± 0.1, 2.1 ± 0.1, 2.4 ± 0.2, 2.3 ± 0.1, and 2.2 ± 0.2 ms for 1 μm, 10 μm, 100 μm, 1 mm, and 30 mm ATP, respectively. The accumulated distribution for all tested [ATP]s is shown at the *bottom*, and the time constant was estimated as 2.5 ± 0.2 ms. *D*, distributions of angle difference (Δθ) from main pause to subpause. The distributions were fitted with Gaussian functions. The peak angles were obtained as 36.8 ± 0.4, 32.8 ± 1.8, 41.7 ± 0.4, 42.5 ± 0.3, 42.0 ± 0.6, and 35.2 ± 0.8° at 1 μm, 10 μm, 100 μm, 1 mm, and 30 mm ATP, respectively. The accumulated distribution for all tested [ATP]s is shown at the *bottom*, and the peak angle was estimated as 39.8 ± 0.1°. *E*, [ATP] dependences of the time constants. The *red circles* and *blue squares* show the time constant of main pause and subpause, respectively. The *red line* indicates the linear fit of τ_1_ from 100 nm to 10 μm ATP. *F*, [ATP] dependence of Δθ. The *orange triangles* show the peak angle of Gaussian fitting at each [ATP].

The rotation velocity of the WT EhV_1_ changed depending on [ATP] and followed Michaelis–Menten kinetics (Fig. 2*A*). The maximum rotation velocity (*V*_max_^ATP^) and the Michaelis constant (*K_m_*^ATP^) were 117 ± 3 revolutions per second (rps; fitted parameter ± fitting error) and 43 ± 6 μm, respectively. The binding rate constant of ATP (*k*_on_^ATP^) determined from 3 × *V*_max_^ATP^/*K_m_*^ATP^ was 8.2 × 10^6^ m^−1^ s^−1^. The *V*_max_^ATP^ was similar to that of the previous study (107 rps; Fig. S1*C*), whereas the *K_m_*^ATP^ was 4 times smaller than that of the previous study (154 μm) (21). As a result, the *k*_on_^ATP^ was 4 times larger than that of the previous study (2.2 × 10^6^ m^−1^ s^−1^). The kinetic parameters obtained are summarized in Table 1.

**Table 1:** **Kinetic parameters of WT and Arg-finger mutant EhV_1_ driven by ATP or ATPγS**

Protein	Substrate	*K_m_^a^*	*V*_max_*^a^*	*k*_on_	Source/Reference
		μ*m*	*rps*	*m*^−*1*^ *s*^−*1*^	
WT EhV_1_ previous construct	ATP	154 ± 33	107 ± 5	2.2 × 10^6^*^b^*	Ref. 21
WT EhV_1_ new construct	ATP	43 ± 6	117 ± 3	8.2 × 10^6^*^b^*	This work
WT EhV_1_ new construct	ATP			1.0 × 10^7^*^c^*	This work
WT EhV_1_ new construct	ATPγS	1.2 ± 0.05	2.1 ± 0.4	1.7 × 10^6^*^b^*	This work
Arg-finger mutant EhV_1_*^d^* new construct	ATP	0.29 ± 0.02	0.38 ± 0.11	2.3 × 10^6^*^b^*	This work

*^a^* The Michaelis constant (*K_m_*) and the maximum velocity (*V*_max_) obtained by fitting of the [ATP] or [ATPγS] dependence of velocity with the Michaelis–Menten equation: *V* = *V*_max_^S^ × [S]/(*K_m_*^S^ + [S]), where S is ATP or ATPγS.

*^b^* The binding rate constant (*k*_on_) for ATP or ATPγS determined from *k*_on_^S^ = 3 × *V*_max_^S^/*K_m_*^S^, where S is ATP or ATPγS.

*^c^* The *k*_on_ for ATP estimated from the time constants of the main pause at 100 nm and 1 μm ATP shown in Fig. 2*B*.

*^d^* A_3_B(R350K)_3_DF complex.

To obtain the time constants and kinetic parameters, we then analyzed the durations for the main pause and subpause at various [ATP]s. The distributions of duration time for the main pause were dependent on [ATP] (Fig. 2, *B* and *E*), whereas those for the subpause were independent of [ATP] (Fig. 2, *C* and *E*). These results indicate that ATP binding occurs during the main pause.

At low [ATP], 100 nm and 1 μm, the distributions of duration time for the main pause were fitted by single exponential decay functions with the time constant (τ_1_) of 972 ± 41 ms and 95.7 ± 3.7 ms, respectively (Fig. 2*B*, *top* and *second from top*). Under these conditions, ATP binding is rate-limiting, and the time constants were inversely proportional to [ATP]. The value of *k*_on_^ATP^ estimated from the time constant and [ATP] (= 1/([ATP] × τ_1_)) was 1.0 × 10^7^ m^−1^ s^−1^ for both 100 nm and 1 μm ATP, consistent with 8.2 × 10^6^ m^−1^ s^−1^ determined from 3 × *V*_max_^ATP^/*K_m_*^ATP^ (Fig. 2*A*).

At high [ATP], 1 mm and 30 mm, the distributions of duration time for the main pause showed convex shapes and were fitted by double exponential decay functions assuming two consecutive first-order reactions (Fig. 2*B*, *second from bottom* and *bottom*; also see “Experimental procedures”). Under this condition, the durations of ATP binding (90–120 μs and 3–4 μs for 1 mm and 30 mm ATP, respectively, estimated from *k*_on_^ATP^) are too short to be detected with the 100 μs time resolution (10,000 fps) of this study. Therefore, two time constants (τ_2_ and τ_3_) of 0.5–0.7 ms, corresponding to the elementary steps other than ATP binding, were obtained.

At intermediate [ATP], 10 and 100 μm, the distributions of duration time for the main pause again showed convex shapes and were fitted by double exponential decay functions assuming two consecutive first-order reactions (Fig. 2*B*, *middle*). The [ATP] dependence of the two time constants obtained is shown in Fig. 2*E*. One of the two time constants obtained at 10 and 100 μm ATP was dependent on [ATP] and consistent with the durations of ATP binding (Fig. 2*E*, plots on the *red line*). Therefore, these time constants were considered to be τ_1_. Another (τ′) was comparable with those obtained at high [ATP]. Therefore, τ′ was considered to include τ_2_ and τ_3_, which could not be separated.

The distributions of duration time for the subpause are shown in Fig. 2*C*. In contrast to those for the main pause, the distributions were independent of [ATP] and almost similar. The distributions at each [ATP] were fitted by the single exponential decay functions with the time constants (τ_4_) of 2.1–2.7 ms. From the fitting of the accumulated distribution for all tested [ATP]s (Fig. 2*C*, *bottom*), the time constant was estimated as 2.5 ± 0.2 ms. Consequently, the three time constants obtained (τ_2_ and τ_3_ at the main pause and τ_4_ at the subpause) will correspond to ATP cleavage, ADP release, or P_i_ release.

We also investigated the angle difference (Δθ) from the main pause to the subpause (Fig. 2*D*). The distributions of Δθ at each [ATP] were similar each other and well-fitted by Gaussians with the peaks around 40° (Fig. 2*F*). From the fitting of the accumulated distribution for all tested [ATP]s (Fig. 2*D*, *bottom*), the peak value of 39.8 ± 0.1° was obtained.

The distributions of duration time for main pause and subpause and the distributions of Δθ for individual molecules at each [ATP] are also shown in Fig. S2. At each [ATP], there were no large differences in the distributions of duration time and Δθ for individual molecules. Therefore, we concluded that the values obtained by our single-molecule analysis with a relatively small sample number (at least 3 molecules per experimental condition) are valid.

### ATPγS-driven rotation and angular position of bound cleavage

The rotation of the WT EhV_1_ driven by ATPγS was observed at various ATPγS concentrations ([ATPγS]s) from 100 nm to 1 mm (Fig. S3). It is known that ATPγS is a nonhydrolyzable or slowly hydrolyzable ATP analog for ATPases associated with various cellular activities (AAA+) proteins (61–66). F_1_ and V_1_ can hydrolyze ATPγS, although the cleavage rate is very low as compared with that of ATP, and the rotation velocity is much lower than that of ATP-driven rotation (20, 23). Rotation velocity of the WT EhV_1_ became much lower than that of ATP-driven rotation. The rotation velocity followed the Michaelis–Menten kinetics, and the *V*_max_^ATPγS^ and *K_m_*^ATPγS^ were 1.2 ± 0.1 rps and 2.1 ± 0.4 μm, respectively (Fig. S3 and Table 1). The *k*_on_^ATPγS^ determined from 3 × *V*_max_^ATPγS^/*K_m_*^ATPγS^ was 1.7 × 10^6^ m^−1^ s^−1^.

**Figure 3.**
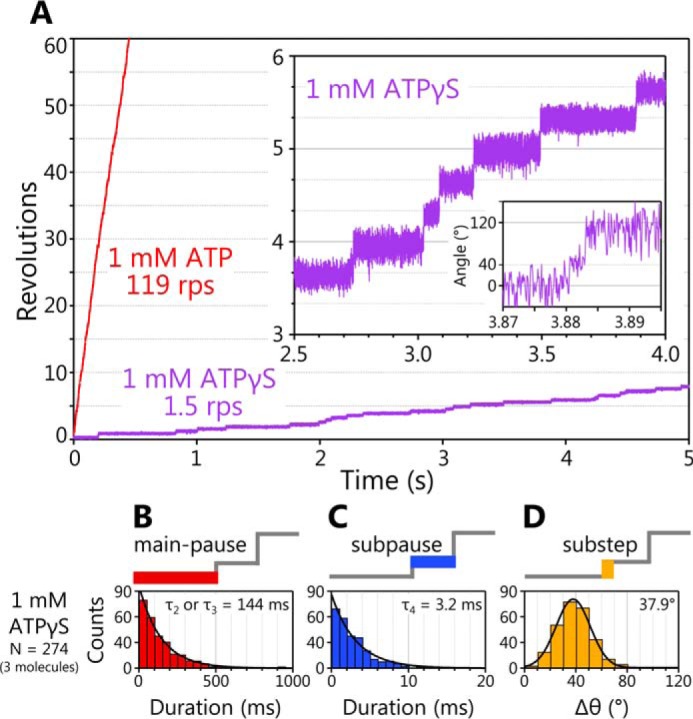
**ATPγS-driven rotation of EhV_1_ and dwell time analysis.** *A*, typical time courses of the rotation of WT EhV_1_ at 1 mm ATPγS. The expanded trace of ATPγS-driven rotation is shown in the *inset*. For comparison, an example of rotation at 1 mm ATP is also shown. *B*, distribution of duration of the main pause in rotation at 1 mm ATPγS. The distribution was fitted with a single-exponential decay function, and the time constant was estimated as 144 ± 3 ms (fitted parameter ± fitting error). *C*, distribution of duration of the subpause in rotation at 1 mm ATPγS. The distribution was fitted with a single-exponential decay function, and the time constant was estimated as 3.2 ± 0.2 ms. *D*, distribution of angle difference (Δθ) from main pause to subpause in rotation at 1 mm ATPγS. The distribution was fitted with a Gaussian function. The peak angle was estimated as 37.9 ± 0.2°. For comparison, the data for rotation at 1 mm ATP are shown in Fig. 2 (*B–D*).

At 1 mm ATP or ATPγS, under the condition that ATP or ATPγS binding is not rate-limiting of rotation, the rotation velocity driven by ATPγS was ∼100 times lower than that driven by ATP (Fig. 3*A*). Even in the case where ATPγS was used, EhV_1_ also showed short subpauses between the main pauses separated by 120° (Fig. 3*A*, *inset*). To verify the pause (main pause or subpause) at which ATPγS cleavage occurs, we analyzed the durations of main pause and subpause at 1 mm ATPγS and compared them with those at 1 mm ATP (Fig. 2, *B* and *C*). As a result, the [ATP]-independent time constant during the main pause (τ_2_ or τ_3_, 144 ms) was much longer than that (0.5–0.7 ms) of ATP-driven rotation (Fig. 3*B*). On the other hand, the τ_4_ in subpause (3.2 ms) and Δθ (37.9°) were similar (2.5 ms and 39.8°) to that of ATP-driven rotation (Fig. 3, *C* and *D*). Therefore, we concluded that ATP cleavage occurs during the main pause, and τ_2_ or τ_3_ corresponds to the time constant for ATP cleavage.

### Backward steps in ATPγS-driven rotation of WT in the presence of ADP

**Figure 4.**
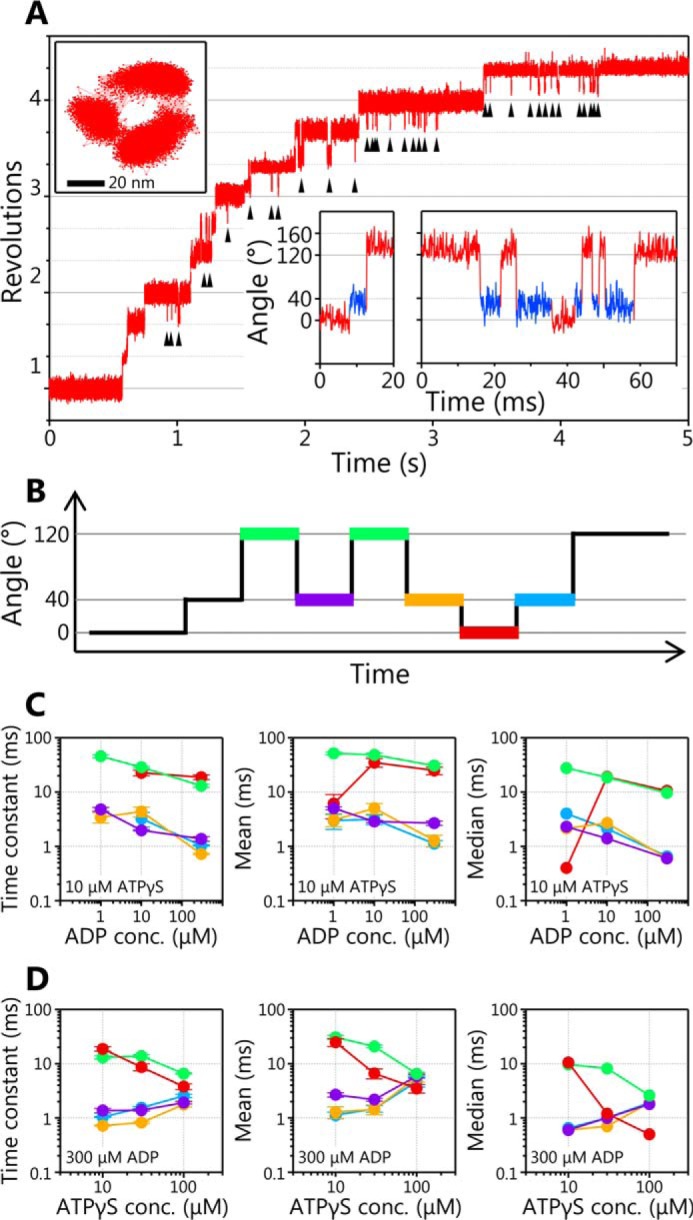
**Backward steps in ATPγS-driven rotation of WT EhV_1_ in the presence of ADP.** *A*, typical time course of EhV_1_ rotation at 10 μm ATPγS in the presence of 100 μm ADP. Backward steps are indicated by *black arrowheads*. The *x-y* trajectory and expanded time courses are shown in *insets*. In the expanded time course, the regions of *red* and *blue* indicate the angular position of the main pause and the subpause, respectively. *B*, the *schematic* of backward and recovery steps. The pauses before the −80° backward step (*green lines*), before the +80° recovery step after the −80° backward step (*purple*), before the −40° backward step (*orange*), before the +40° recovery step (*red*), and before the +80° recovery step after the +40° recovery step (*blue*) are shown. *C*, [ADP] dependences of time constants, mean and median values of the pauses shown in *B* at 10 μm ATPγS in the presence of 1, 10, and 100 μm ADP. *D*, [ATPγS] dependences of time constants, mean and median values of the pauses shown in *B* at 10, 30, and 100 μm ATPγS in the presence of 300 μm ADP. The distributions of pause durations are shown in Fig. S4. *Error bars*, S.D.

When ATPγS-driven rotations of the WT EhV_1_ were observed, we occasionally found that the addition of ADP causes frequent backward steps during the rotation. A typical example of backward steps during the rotation at 10 μm ATPγS in the presence of 100 μm ADP is shown in Fig. 4*A* (*arrowheads* and *insets*). The backward steps were observed only in the presence of ADP and in the rotation where the hydrolysis rate is slowed down by ATPγS. Furthermore, two kinds of backward steps were observed. One is the backward step from the main pause to the previous subpause position (−80° backward step), and another is the occasional further backward step to the previous main pause position (−80° and subsequent −40° backward steps). However, multiple backward steps larger than −120° were not observed.

To elucidate the chemo-mechanical coupling mechanism of the backward step, we analyzed the durations of the pauses before −80° backward steps (Fig. 4*B*, *green*), before +80° recovery steps after −80° backward steps (*purple*), before −40° backward steps (*orange*), before +40° recovery steps (*red*), and before +80° recovery steps after +40° recovery steps (*blue*) at various [ADP]s or [ATPγS]s (Fig. S4). The time constants were estimated from the fitting of the single exponential decay functions. In addition, the mean and median values were calculated because some distributions were not well-fitted by the single exponential decay functions due to an insufficient number of events.

When [ADP] dependences were compared (Fig. 4*C*), the time constants, means, and medians for all five kinds of pauses (Fig. 4*B*) slightly decreased with increase of [ADP], but they were not strongly dependent on [ADP]. On the other hand, when [ATPγS] dependences were compared (Fig. 4*D*), those for pauses before −80° backward steps (Fig. 4*D*, *green*) and before +40° recovery steps (Fig. 4*D*, *red*) slightly decreased with increase of [ATPγS], but those for the other three pauses slightly increased with increase of [ATPγS] (Fig. 4*D*). Among them, the pause before +40° recovery steps showed the largest dependence on [ATPγS]. This result implies that ATPγS binding triggers a +40° recovery step. However, the details of the chemo-mechanical coupling mechanism remain unclear. Therefore, we tried to observe the backward steps without using ATPγS.

### Backward steps in ATP-driven rotation of Arg-finger mutant in the presence of ADP

**Figure 5.**
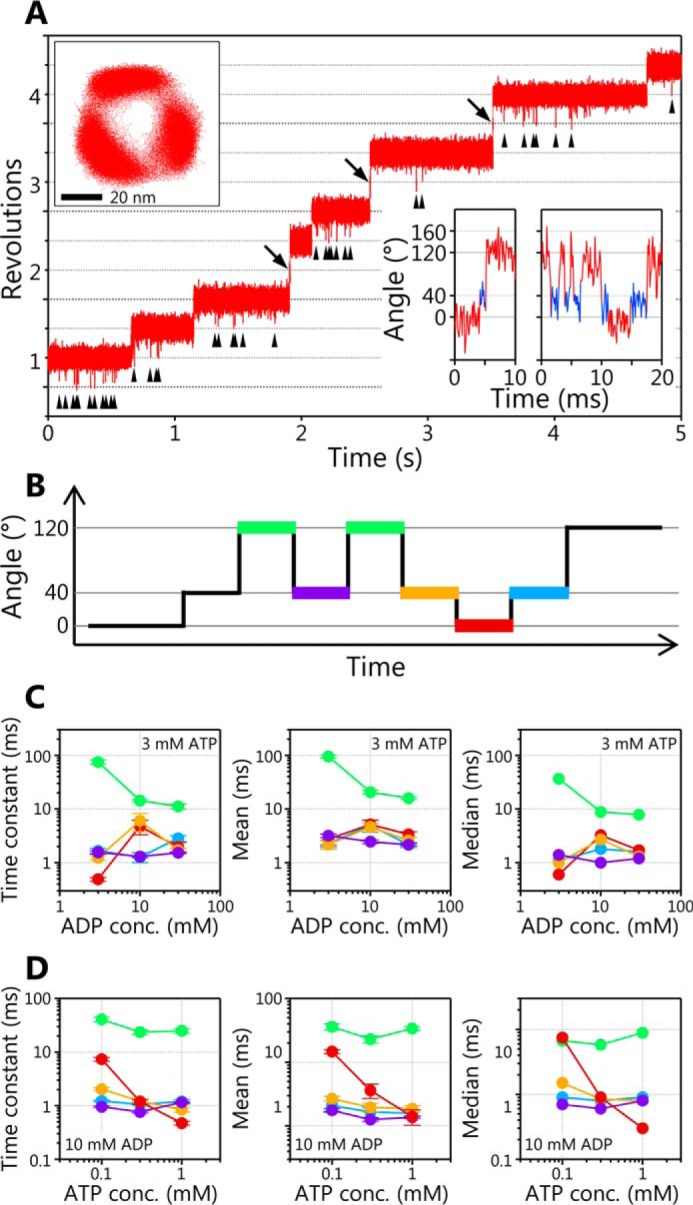
**Backward steps in ATP-driven rotation of Arg-finger mutant EhV_1_ in the presence of ADP.** *A*, typical time course of rotation at 3 mm ATP in the presence of 10 mm ADP. The *black arrowheads* indicate the backward steps, and the *black arrows* indicate the 240° forward steps due to the alternative pathways as observed in TF_1_. The *x-y* trajectory and the expanded time course are shown in *insets*. In the expanded time course, the regions of *red* and *blue* indicate the angular position of the main pause and the subpause, respectively. *B*, the *schematic* of backward and recovery steps. The pauses before the −80° backward step (*green lines*), before the +80° recovery step after the −80° backward step (*purple*), before the −40° backward step (*orange*), before the +40° recovery step (*red*), and before the +80° recovery step after the +40° recovery step (*blue*) are shown. *C*, [ADP] dependences of time constants, mean and median values of the pauses shown in *B* at 3 mm ATP in the presence of 3, 10, and 30 mm ADP. *D*, [ATP] dependences of time constants, mean and median values of the pauses shown in *B* at 0.1, 0.3, and 1 mm ATP in the presence of 10 mm ADP. The distributions of pause durations are shown in Fig. S5. *Error bars*, S.D.

We found that the backward steps were also observed in ATP-driven rotation of an Arg-finger mutant EhV_1_ (A_3_B(R350K)_3_DF complex) in the presence of ADP (*arrowheads* and *insets* in Fig. 5*A*). The Arg-finger is a catalytic arginine residue that plays a role in stabilization of the transition state and acceleration of the hydrolysis rate in ATPases (58–60). In the biochemical assay, the Arg-finger mutant EhV_1_ showed very low ATPase activity compared with the WT (16). In the presence of ADP, ATP-driven rotation of the Arg-finger mutant EhV_1_ showed two kinds of backward step (−80 and −40° backward steps), the same as ATPγS-driven rotation of WT EhV_1_.

Then we analyzed the durations of the five kinds of pauses (Fig. 5*B*) at various [ADP]s or [ATP]s (Fig. S5). The time constants were estimated by the fitting of single exponential decay functions. In addition, the mean and median values were calculated. When [ADP] dependences were compared (Fig. 5*C*), only the time constants for the pauses before −80° backward steps (*green*) clearly decreased with increase of [ADP], although those for the other four pauses were not dependent on [ADP]. The means and medians for the pauses before −80° backward steps showed less clear [ADP] dependences than that of time constants, presumably due to the difference in the number of minor short durations at each [ADP] (Fig. S5*A*). When [ATP] dependences were compared (Fig. 5*D*), on the other hand, only the time constants, means, and medians for pauses before +40° recovery steps (*red*) clearly decreased with increase of [ATP], whereas those for the other four pauses were not dependent on [ATP]. From these results, we concluded that −80° backward and +40° recovery steps of the Arg-finger mutant EhV_1_ are triggered by ADP and ATP bindings, respectively.

Interestingly, in the ATP-driven rotation of the Arg-finger mutant EhV_1_, we also often observed rapid 240° forward steps (*arrows* in Fig. 5*A*), suggesting the presence of alternative reaction pathways as observed in TF_1_ (67). Detailed analysis of the alternative reaction pathways of the Arg-finger mutant EhV_1_ will be published elsewhere.

### Rotation in high concentration of P_i_

**Figure 6.**
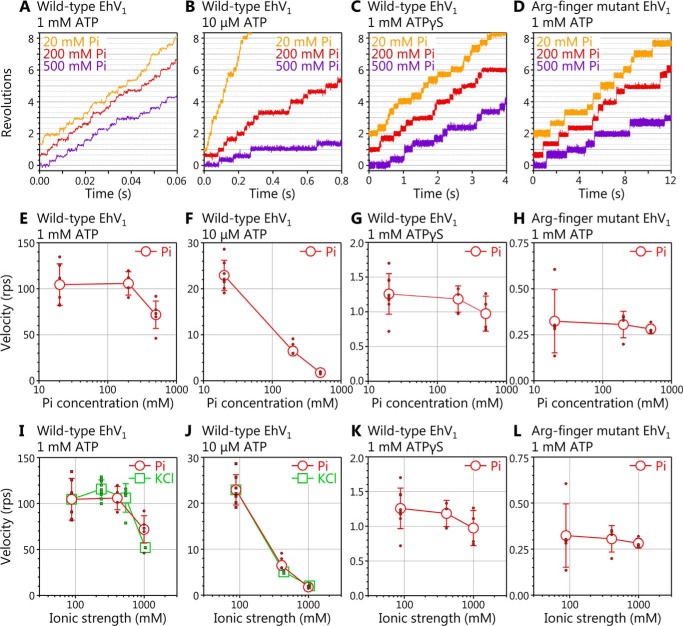
**Rotation of WT and Arg-finger mutant EhV_1_ in the presence of a high concentration of P_i_.** *A–D*, examples of time course of the rotation. The rotations of WT EhV_1_ at 1 mm ATP (*A*), 10 μm ATP (*B*), 1 mm ATPγS (*C*), and Arg-finger mutant EhV_1_ at 1 mm ATP (*D*) were observed at 10,000 fps in the presence of 20, 200, and 500 mm P_i_. *E–H*, P_i_ concentration dependences of rotation velocity. The *red open circles* and *small filled circles* indicate average velocity and velocities of individual molecules in the presence of P_i_, respectively. *I–L*, ionic strength dependences of rotation velocity. The *red open circles* and *small filled circles* indicate average velocity and velocities of individual molecules in the presence of P_i_, respectively. The *green open squares* and *small filled squares* in *I* and *J* indicate average velocity and velocities of individual molecules in the presence of KCl, which is added instead of P_i_ as an ion source. *Error bars*, S.D.

To confirm whether backward steps triggered by P_i_ binding are observed, rotation of the WT EhV_1_ driven by ATP or ATPγS and that of the Arg-finger mutant EhV_1_ driven by ATP were observed in high P_i_ concentration ([P_i_]) (Fig. 6, *A–D*). At 1 mm ATP, the velocity of the WT decreased ∼40% in 500 mm P_i_ (which corresponds to ionic strength of 994 mm) as compared with that in 20 mm P_i_ (which corresponds to ionic strength of 88 mm) (Fig. 6*E*). However, backward steps were not observed. Moreover, velocity decrement was not specific to P_i_ because the velocity also decreased to a similar level in the presence of 1000 mm KCl (ionic strength of 1038 mm, similar to that of 500 mm P_i_) (Fig. 6*I*). At 10 μm ATP, the velocity of WT EhV_1_ largely decreased with the increase of [P_i_] (Fig. 6*F*). However, again, the backward steps were not observed, and the velocity decrement was not specific to P_i_ because KCl also caused large velocity decrement (Fig. 6*J*). These results indicate that there are no P_i_-specific inhibitions in the ATP-driven rotation of the WT EhV_1_. Instead, high ionic strength likely causes the decrease of rotation velocity by decreasing the ATP binding rate and/or by destabilizing the EhV_1_ complex.

The backward steps were also not observed in the rotation of the WT driven by 1 mm ATPγS and that of the Arg-finger mutant driven by 1 mm ATP in high [P_i_]. In contrast to the ATP-driven rotation of the WT, the rotation velocity did not largely change, depending on [P_i_] or ionic strength (Fig. 6, *G*, *H*, *K*, and *L*), presumably because rotation velocity is already much lower than that of the ATP-driven rotation of the WT.

## Discussion

We previously reported that the EhV_1_ has two distinct reversible rotational states, namely clear and unclear, and concluded that the unclear rotation is caused by the unstable interaction between the stator A_3_B_3_ ring and the rotor DF subcomplex of the EhV_1_ (21, 55). However, in the present study, we prepared a new construct of EhV_1_ with minimum additional amino acid residues in the rotor DF (Fig. S1) and found that the newly constructed EhV_1_ showed only clear rotation (Fig. 1, *A–C*). This result strongly suggests that the unclear rotation observed in the previous study is caused by the additional amino acid residues in the DF. In other words, the unstable interaction between the stator A_3_B_3_ and the rotor DF is caused by these additional amino acid residues. Interestingly, the *k*_on_^ATP^ for new construct increased 4 times as compared with that of the previous one (Fig. S1 and Table 1). This result strongly suggests that ATP binding is facilitated by the increased stability of the new construct.

In addition to the persistent clear rotation, we found that the EhV_1_ shows a subpause that divides the 120° step into 40 and 80° substeps (Fig. 1, *D* and *E*). In the ATP-driven rotation of WT EhV_1_, three time constants (τ_1_, τ_2_, and τ_3_) in the main pause and a time constant (τ_4_) in the subpause were obtained from the analysis of duration times (Fig. 2, *B* and *C*). Because the τ_1_ was inversely proportional to [ATP] in the range of 100 nm to 10 μm ATP at which ATP binding is rate-limiting (Fig. 2*E*), we concluded that ATP binding occurs during the main pause, and τ_1_ corresponds to the time constant for ATP binding. Other time constants (τ_2_, τ_3_, and τ_4_) with the length of a few milliseconds were not dependent on [ATP], indicating that these time constants correspond to the elementary steps of the ATP hydrolysis reaction other than ATP binding, namely ATP cleavage, ADP release, or P_i_ release. The subpause was observed not only in ATP-driven rotation but also in ATPγS-driven rotation of the WT (Fig. 3*A*). From the analysis of duration times, τ_2_ or τ_3_ in the main pause of ATPγS-driven rotation was much longer than that of ATP-driven rotation, whereas τ_4_ in subpause was similar (Fig. 3, *B* and *C*). Therefore, we concluded that ATP cleavage occurs during main pause, and τ_2_ or τ_3_ corresponds to the time constant for ATP cleavage.

In the presence of ADP, the WT EhV_1_ showed frequent backward steps in the main pause during ATPγS-driven rotation (Fig. 4*A*). There were two kinds of backward steps, the −80° backward step from the main pause to the previous subpause and an occasional further −40° backward step after the −80° backward step. Similar backward steps were also observed in ATP-driven rotation of the Arg-finger mutant EhV_1_ in the presence of ADP (Fig. 5*A*). From the results of detailed analysis of duration times before and after backward steps (Figs. 4 and 5 and Figs. S4 and S5), we concluded that −80° backward and 40° recovery steps are triggered by ADP and ATP (ATPγS) bindings, respectively. If we assume that recovery steps are the reverse reactions of backward steps, it is considered that +80° recovery and −40° backward steps are triggered by ADP and ATP (ATPγS) releases, respectively. In addition, assuming that recovery steps correspond to normal forward steps, we concluded that 40 and 80° substeps are triggered by ATP (ATPγS) binding and ADP release, respectively. Therefore, τ_4_ in the subpause corresponds to the time constant for ADP release.

On the other hand, even in the presence of very high concentrations of P_i_, the backward steps and the decrease of velocity specific to P_i_ were not observed in rotation of the WT driven by ATP or ATPγS and that of the Arg-finger mutant driven by ATP (Fig. 6). These results strongly suggest that the affinity of P_i_ to the catalytic site of EhV_1_ is very low, and P_i_ easily dissociates from the catalytic site after ATP cleavage, and/or P_i_ present in solution does not bind to the catalytic site easily. Therefore, we concluded that τ_2_ or τ_3_ in the main pause corresponds to the time constant for P_i_ release.

At high [ATP], in the main pause, we assumed the reaction scheme without reverse reaction (ATP synthesis) for analysis (see Scheme 1 under “Experimental procedures”). However, if ATP synthesis occurs on the catalytic site, we should consider another reaction scheme including the reverse reaction (see Scheme 2). With Scheme 2, we obtained the values of time constants for ATP cleavage (τ_hyd_^ATP^ in Equation 2 under “Experimental procedures”), ATP synthesis (τ_syn_^ATP^), and P_i_ release (τ_off_^Pi^) as 0.7, 0.7, and 1.8 ms, respectively. Furthermore, by comparing τ_syn_^ATP^ (0.7 ms) and τ_off_^Pi^ (1.8 ms), the probability of reverse reaction (ATP synthesis) and the expected number of reverse reactions were estimated as 72% and 2.6 per single main pause, respectively. It has been reported that under the unisite catalysis condition, where ATP binds to only one of three catalytic sites, F_1_ shows repetitive rapid interconversion between ATP and ADP + P_i_ states (68–70). However, increment of [ATP] prevents the reversal of ATP hydrolysis (69, 71), indicating that the repetitive interconversion of ATP/ADP + P_i_ does not occur under the multisite catalysis condition in which F_1_ rotates unidirectionally, driven by ATP hydrolysis. In this study, we employed Scheme 1 rather than Scheme 2, because multisite catalysis occurs during unidirectional rotation of EhV_1_, and the multiple rounds of ATP cleavage/synthesis were also not considered in the previous single-molecule studies of F_1_ and V_1_ (19–29, 31–35).

**Figure 7.**
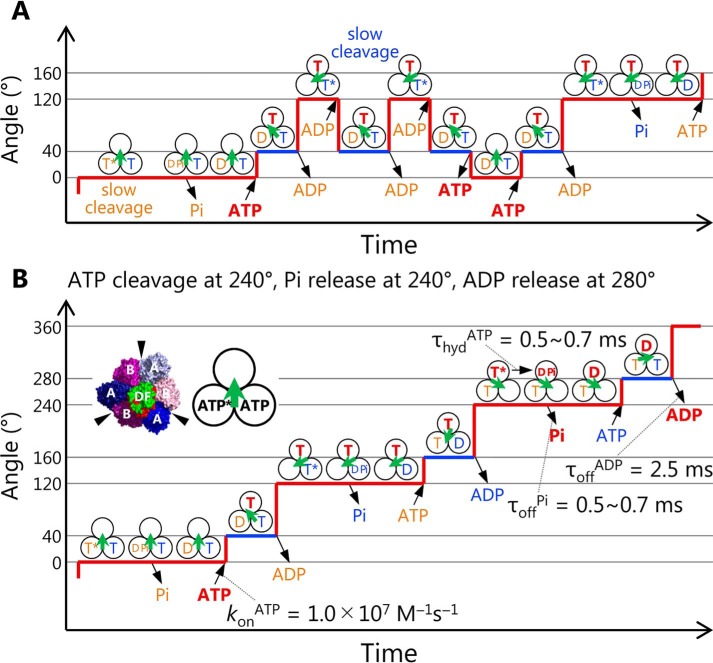
**A model of chemo-mechanical coupling of EhV_1_ including substeps and backward steps.** *A*, model of backward steps described with the nucleotide states of three catalytic sites. *Three black circles* and a *green arrow* indicate the catalytic sites and the direction of rotor DF of EhV_1_, respectively. Under the conditions where bond cleavage durations are very long, such as ATPγS-driven rotation of WT or ATP-driven rotation of Arg-finger mutant, EhV_1_ shows backward steps in the presence of ADP. There are two kinds of the backward step, the −80° backward step from the main pause (*red line*) to the previous subpause (*blue line*) and the subsequent −40° backward step to the previous main pause. In our model, the −80 and −40° backward steps are triggered by ADP binding and ATP release, respectively. Also, the 40 and 80° recovery steps are triggered by ATP binding and ADP release, respectively. *B*, chemo-mechanical coupling scheme of EhV_1_ in normal rotation. During the main pause, ATP cleavage occurs, and P_i_ is released immediately. Then ATP binds to the empty site, and rotor DF rotates 40°. During the subpause, product ADP is released, and then the rotor DF subcomplex rotates 80°. In this scheme, when we focus on the single catalytic site on top, ATP (shown in *red*) binds at 0°, and then bound ATP is cleaved into ADP and P_i_ at 240°, P_i_ is released at the same angle, and ADP is released at 280°. In our model, the previous crystal structure corresponds to a state during the main pause.

Fig. 7*A* shows a model of a chemo-mechanical coupling scheme for the backward step of EhV_1_. It is worth noting that the backward step in the presence of ADP was observed only when the duration time of ATP cleavage was prolonged by using ATPγS or the Arg-finger mutant, which is very different from the physiological condition. Therefore, we expect that the backward steps do not occur under physiological conditions. On the other hand, the observation of backward step is important to consider the reversibility of ATP hydrolysis/synthesis reaction of EhV_1_. Our results suggest the possibility that EhV_1_ can synthesize ATP if it is forcibly rotated in the opposite direction, as reported for TF_1_ (72). Furthermore, interestingly, we also found that the backward steps occur in the early part of the main pauses (Fig. S6). This result suggests that ADP binding to an empty catalytic site can occur during main pause before ATP cleavage in another catalytic site.

Fig. 7*B* shows a model of the chemo-mechanical coupling scheme of EhV_1_ rotation based on the present single-molecule study and the previous structural information. If we consider only the results of single-molecule analysis, eight models can be possible (Fig. S7). They are roughly classified into three models: after ATP binding at 0°, ATP cleavage at 120°, and ADP release at 160° (Fig. S7*A*); ATP cleavage at 120° and ADP release at 280° (Fig. S7*B*); and ATP cleavage at 240° and ADP release at 280° (Fig. S7*C*). Each model has two or three submodels with different timings of P_i_ release. In these models, EhV_1_ can bind with one or two or with two or three nucleotides (ATP or ADP) during the catalytic cycles. On the other hand, in the previous crystal structures (16, 17), it has been shown that EhV_1_ bound with two or three nucleotides. This structural information restricts possible models to those shown in Fig. S7 (*B* and *C*). Furthermore, if we compare catalytic states of the remaining models with those of the “catalytic dwell” crystal structure (empty, tight, bound in the counterclockwise direction; Fig. S7*D*) (16, 17), we can further restrict possible models to the two shown in Fig. S7*C*. Finally, considering the low affinity of P_i_ (Fig. 6), we prefer the model shown in Fig. 7*B*, in which ATP is cleaved at 240°, P_i_ is released at 240° (immediately after the ATP cleavage into ADP and P_i_), and ADP is released at 280°.

It is worth noting that in the previous structural analysis, there were no differences in the rotational angles of the rotor DF between “catalytic dwell” and “ADP release dwell” crystal structures, in which EhV_1_ binds with two and three nucleotides, respectively (16, 17). On the other hand, in our chemo-mechanical coupling model of EhV_1_ (Fig. 7*B*), the rotational angle of the rotor DF for the ADP release dwell at the subpause is 40° ahead from that for the catalytic dwell at the main pause. In the crystal structure of the ADP release dwell, all three catalytic sites of EhV_1_ bind with ADP. On the other hand, in our model, two catalytic sites bind with ATP, and one binds with ADP during the ADP release dwell at the subpause. We consider that difference of the nucleotides bound to the three catalytic sites is relevant to the apparent discrepancy of rotational angle of the rotor DF between the crystal structure and our model.

In this study, we have shown that EhV_1_ also shows a substep like F_1_, although the mechanism of chemo-mechanical coupling is largely different from those of F_1_. In F_1_, although the angle and the number of substeps are different between TF_1_ and HF_1_ (22–29), the elementary steps that generate a substep are similar to each other; the first substep is triggered by ATP binding coupled with ADP release, the second substep is triggered by P_i_ release, and the third substep for HF_1_ is triggered by ATP cleavage. On the other hand, in EF_1_, it has been reported that ADP is released at around 50° after ATP binding during the first substep, although the pause duration waiting for ADP release has not been estimated (35). According to our chemo-mechanical coupling scheme of EhV_1_ (Fig. 7*B*), ATP binding and ADP release occur at different angles and trigger substeps separately as EF_1_. In addition, in our model, ATP cleavage in one catalytic site and ATP binding to another empty catalytic site occur at the same angle, similar to TtV_1_ (20) and A_1_-ATPase from *Methanosarcina mazei* A-type ATP synthase (73). Therefore, it is suggested that this similarity comes from structural similarity, not from physiological function.

In F_1_, it has also been proposed that ADP is released prior to P_i_. If P_i_ is occasionally released prior to ADP, TF_1_ lapses into the so-called MgADP-inhibited state and stops rotation (28). On the other hand, in our chemo-mechanical coupling scheme of EhV_1_ (Fig. 7*B*), P_i_ release occurs before ADP release and does not trigger a substep, although the timing of P_i_ release has not been completely determined in the present study. In the previous single-molecule manipulation of TF_1_, it has also been proposed that the order of ADP and P_i_ release (ADP first, P_i_ second) is important for efficient ATP synthesis in the cell in which [ATP] is much higher than [ADP] (27). For selective binding of ADP instead of ATP, the catalytic site must be occupied with P_i_ in advance. Considering the physiological function of EhV-ATPase as an ion pump driven by ATP hydrolysis, EhV_1_ does not have to release ADP prior to P_i_. In this aspect, evaluation of the ATP synthesis ability of EhV-ATPase driven by the electrochemical potential gradient of the ion across the cell membrane will be an important issue to be addressed, to understand reversibility of energy conversion and functional differentiation of the rotary ATPases.

## Experimental procedures

### Purification of proteins

EhV_1_ was reconstituted from A_3_B_3_ and biotinylated DF subcomplexes. The A_3_B_3_ and DF subcomplexes were prepared separately to biotinylate only DF subcomplex. The WT A_3_B_3_, Arg-finger mutant A_3_B(R350K)_3_, and DF were expressed in *E. coli* BL21 (DE3) cells from the expression plasmids pTR19-A(His_6_ at N)B, pTR19-A(His_6_ at N)B(R350K), and pTR19-D(Riken-His at N/M1G/T60C/R131C)F(Riken-His at N). Cells were transformed with expression plasmid and cultivated in Super broth (32 g/liter tryptone, 20 g/liter yeast extract, and 5 g/liter NaCl) containing 100 μg/ml ampicillin and 1 mm isopropyl-β-d-thiogalactopyranoside at 37 °C overnight. Cells were suspended in Equilibrium buffer (20 mm KP_i_ (pH 8.0), 230 mm NaCl, and 20 mm imidazole) and disrupted by sonication. The cell debris was separated by centrifugation (81,000 × *g*, 20 min, 4 °C), and then the soluble fraction was applied to a nickel-nitrilotriacetic acid column (Ni-NTA Superflow, Qiagen) equilibrated with Equilibrium buffer. After washing with 10 column volumes of Equilibrium buffer, the A_3_B_3_, A_3_B(R350K)_3_, or DF was eluted with Elution buffer (20 mm KP_i_ (pH 8.0), 50 mm NaCl, and 200 mm imidazole). The eluted fractions were concentrated with a centrifugal concentrator (Amicon Ultra-10K, Merck Millipore). Then the A_3_B_3_ or A_3_B(R350K)_3_ passed through a gel-filtration column (Superdex 200, GE Healthcare) equilibrated with Gel-filtration buffer A (20 mm MES-NaOH (pH 6.5), 100 mm NaCl, and 10% glycerol). The purified A_3_B_3_ or A_3_B(R350K)_3_ was flash-frozen in liquid nitrogen and stored at −80 °C before use.

In the case of the DF, TEV-protease treatment was applied to cut Riken-His tag. The concentrated fraction was diluted 5-fold with the TEV treatment buffer (20 mm KP_i_ (pH 8.0), 50 mm NaCl, 0.04% 2-mercaptoethanol, 40 μg/ml TEV protease). After treatment for 16 h at 4 °C, the solution was applied to Ni-NTA Superflow. The flow-through was concentrated with Amicon Ultra-10K, and then the DF was further purified by passing through a Superdex 75 column equilibrated with Gel-filtration buffer B (20 mm Tris-HCl (pH 8.0), 100 mm NaCl). The purified DF was applied to a buffer exchange column (NAP-5 Columns, GE Healthcare) equilibrated with Biotinylation buffer (20 mm KP_i_ (pH 7.0), 150 mm NaCl). The eluted fraction with Biotinylation buffer was concentrated with a centrifugal concentrator (Vivaspin 500, 5000 molecular weight cutoff polyethersulfone, Sartorius) and diluted with Biotinylation buffer including a 3-fold molar excess of biotinylation reagent (biotin-PEAC_5_-maleimide, Dojindo). After incubation for 30 min at 25 °C, the biotinylation was quenched by using 10 mm DTT at 25 °C for 10 min. Then the biotinylated DF was flash-frozen in liquid nitrogen and stored at −80 °C before use.

To reconstitute EhV_1_, purified A_3_B_3_ or A_3_B(R350K)_3_ and biotinylated DF were mixed gently at a 1:2 molar ratio and incubated for 3 h at 25 °C. Reconstituted EhV_1_ was purified by using a Superdex 200 column equilibrated with Gel-filtration buffer A. The purified EhV_1_ was flash-frozen in liquid nitrogen and stored at −80 °C before use. The degrees of biotinylation for WT and Arg-finger mutant EhV_1_ were estimated to be 172 and 178%, respectively, by using the Pierce^TM^ Fluorescence Biotin Quantitation Kit (Thermo Fisher Scientific) with WT EhV_1_ without biotinylation as a negative control. These values are reasonable because two cysteine residues (T60C and R131C) were introduced into the D subunit for biotinylation, and the ideal maximum value is 200%.

The ADP-dependent glucokinase from hyperthermophilic archaea *Pyrococcus furiosus* (PfGK) is an enzyme that phosphorylates glucose using ADP and produces glucose 6-phosphate and AMP (74–77). In the observation of ATPγS-driven rotation of EhV_1_, PfGK and glucose were used to remove the contaminated ADP in ATPγS (Roche Applied Science) as an ADP-quenching system (20). The PfGK was expressed in *E. coli* Tuner (DE3) cells from the expression plasmids pET27b-PfGK. Cells were transformed with expression plasmid and cultivated in LB medium (1% Tryptone, 0.5% g/liter yeast extract, and 1% NaCl) containing 100 μg/ml ampicillin and 1 mm isopropyl-β-d-thiogalactopyranoside at 37 °C overnight. Cells were suspended in 100 mm Tris-HCl (pH 8.0) including 1 mm DTT and disrupted by sonication, and then the cell debris was separated by centrifugation (15,000 × *g*, 30 min). The soluble fraction was heat-treated (90 °C, 30 min), and denatured and aggregated proteins were separated by centrifugation (15,000 × *g*, 30 min). Then heating and centrifugation were repeated twice. The soluble fraction was then passed through a Superdex 200 column equilibrated with 100 mm Tris-HCl (pH 8.0). The purified protein was stored at 4 °C before use.

### Preparation of gold nanoparticles

To 1 ml of 40-nm gold nanoparticle suspension (BBI Solutions), 20 μl of 10% Tween 20 was added and mixed well. Then 20 μl of 1 m KP_i_ (pH 8.0) and 100 μl of Biotinylation solution (1 mg/ml biotin-EG3-undecanethiol (SensoPath Technologies), 2% (v/v) carboxy-EG6-undecanethiol (Dojindo), 2% (v/v) hydroxy-EG6-undecanethiol (Dojindo) dissolved in ethanol) were added and mixed. After incubation at 70 °C overnight, the suspension was centrifuged at 15,000 rpm for 5 min at 25 °C. The pellet was resuspended in 1 ml of Wash buffer (10 mm KP_i_ (pH 8.0), 0.2% Tween 20) and centrifuged again. Then resuspension and centrifugation were repeated six times, and the unreacted biotin was removed. The pellet was resuspended in 1 ml of Wash buffer including 1 mg/ml streptavidin (ProSpec) and mixed by inversion for 3 h at 25 °C. The unreacted streptavidin was removed by repeating centrifugation (15,000 rpm, 5 min, 25 °C) and resuspension with 1 ml of Wash buffer six times. Finally, the pellet was suspended with 100 μl of 10 mm KP_i_ (pH 8.0) and stored at 4 °C before use.

### Single-molecule imaging of rotation

To visualize the rotation of EhV_1_, streptavidin-coated 40-nm gold nanoparticle was attached to biotinylated DF subcomplex as a low-load probe. The rotation of EhV_1_ was observed as a motion of gold nanoparticle in the flow cell. The flow cell was prepared by covering an untreated coverglass (18 × 18 mm^2^, Matsunami Glass) on a coverglass (24 × 32 mm^2^, Matsunami Glass) immersed overnight in piranha solution (H_2_SO_4_/H_2_O_2_ = 3:1) and washed with 1 m KOH for 30 min. By placing four greased spacers between two coverglasses, three chambers with ∼4-mm width and ∼50-μm thickness were arranged side by side. The EhV_1_ of 5–10 nm in Observation buffer A (20 mm KP_i_ (pH 7.0), 230 mm NaCl) was infused into the flow cell and incubated for 10 min. The EhV_1_ molecules were attached to the glass surface by the electrostatic interaction between positively charged histidine tag (His_6_ tag) introduced into the A subunits and negatively charged glass surface. After buffer exchange with 40 μl (∼10 times chamber volume) of Observation buffer A including 5 mg/ml BSA and incubation for 5 min, streptavidin-coated 40-nm gold nanoparticles suspended in Observation buffer A including 5 mg/ml BSA were infused and incubated for 10 min. To remove the unbound gold nanoparticles, the flow cell was washed with 40 μl of Observation buffer B (20 mm KP_i_ (pH 6.5), 50 mm KCl, 2 mm MgCl_2_). The fractions of EhV_1_ molecules on the glass surface bound with gold nanoparticles were estimated to be 0.97 and 0.88% for WT and Arg-finger mutant EhV_1_, respectively, by comparing the density of Cy3-labeled EhV_1_ bound on the glass surface (0.34 molecules/μm^2^ at 50 pm EhV_1_ input, measured by single-molecule fluorescence imaging) and the density of gold nanoparticle attached to the glass surface (0.33 and 0.30 particles/μm^2^ for WT and Arg-finger mutant at 5 nm EhV_1_ input, respectively). After buffer exchange with Observation buffer B containing the prescribed concentration of Mg-ATP and ATP-regenerating system (2.5 mm phosphoenolpyruvate and 0.1 mg/ml pyruvate kinase), observation of rotation was started. When ATPγS was used, the ADP-quenching system (10 mm glucose and 0.1 mg/ml PfGK) (20) was applied instead of the ATP-regenerating system. When ADP was also added, the ATP-regenerating system and ADP-quenching system were omitted.

The gold nanoparticle was observed using an objective-type total internal reflection dark-field microscope (78–80) constructed on an inverted microscope (IX-70, Olympus). The gold nanoparticles were illuminated by the evanescent field, which has a penetration depth of ∼100 nm from the surface of the coverglass. The scattering images of a rotating gold nanoparticle were recorded as an 8-bit movie file using a high-speed CMOS camera (FASTCAM 1024PCI, Photron) at 10,000 fps with a pixel size of 92 nm/pixel. The ratios of rotating gold nanoparticles to all gold nanoparticles bound on the glass surface were 1.4% for WT and 5.4% for Arg-finger mutant EhV_1_. The centroid and rotary angle at each frame of the image sequence were analyzed by a custom-made plugin of the ImageJ software (National Institutes of Health), and the pauses in rotation trajectory were manually identified by eye. For detailed analysis, we selected the EhV_1_ molecules showing similar pausing durations for three main pauses (EhV_1_ molecules rotating “symmetrically”). Due to local roughness of the glass surface, the axis of rotation of EhV_1_ can deviate from the normal direction of the sample plane of the optical microscope. In this case, the gold nanoparticle attached to EhV_1_ would show longer pauses at a specific angle due to increased nonspecific interaction with the glass surface. The rotating molecules for detailed analysis were manually selected by eye.

### Fitting of distributions of duration time for main pause of WT EhV_1_ at high [ATP]

In our study, at high [ATP], the distributions of duration time for the main pause of WT EhV_1_ (Fig. 2*B*) were fitted by double-exponential decay functions assuming two consecutive first-order reactions with two time constants,

(Eq. 1) $$y=A\times \left(e^{-\frac{t}{\tau }}-e^{-\frac{t}{\tau \prime }}\right)$$

where *A* is constant, τ and τ′ are time constants (or *k* = 1/τ, rate constant), and *t* is duration time. According to the model of chemo-mechanical coupling of EhV_1_ (Fig. 7*B*), the reaction scheme for main pause is described as follows,

$$\begin{array}{c} AB_{\text{tight}}\cdot \text{ATP}\overset{k_{\text{hyd}}^{\text{ATP}}}{\to }AB_{\text{tight}}\cdot \left(\text{ADP}+{\text{P}}_{\text{i}}\right)\overset{k_{\text{off}}^{{\text{P}}_{\text{i}}}}{\to }AB_{\text{tight}}\cdot \text{ADP}+{\text{P}}_{\text{i}}\\ \text{SCHEME}\;1 \end{array}$$

where *AB*_tight_ is one of three catalytic sites at which ATP is about to be cleaved. With Scheme 1, two time constants (0.5 and 0.7 ms; Fig. 2*B*, 30 mm ATP) corresponding to ATP cleavage (τ_hyd_^ATP^ = 1/*k*_hyd_^ATP^) or phosphate release (τ_off_^Pi^ = 1/*k*_off_^Pi^) were obtained.

If multiple rounds of ATP cleavage (hydrolysis) and synthesis occur during single main pause, as is the case for unisite catalysis of F_1_ (68–70), the reaction is described with Scheme 2 below.

$$\begin{array}{c} AB_{\text{tight}}\cdot \text{ATP}\overset{k_{\text{hyd}}^{\text{ATP}}}{\underset{k_{\text{syn}}^{\text{ATP}}}{⇄}}AB_{\text{tight}}\cdot \left(\text{ADP}+{\text{P}}_{\text{i}}\right)\overset{k_{\text{off}}^{{\text{P}}_{\text{i}}}}{\to }AB_{\text{tight}}\cdot \text{ADP}+{\text{P}}_{\text{i}}\\ \text{SCHEME}\;2 \end{array}$$

For Scheme 2, the fitting function for the distribution of duration time for the main pause is expressed as follows.

(Eq. 2) $$y=A\times \left(e^{-\frac{t}{\tau_{\text{hyd}}^{\text{ATP}}}}-e^{-\left(\frac{1}{\tau_{\text{syn}}^{\text{ATP}}}+\frac{1}{\tau_{\text{off}}^{{\text{P}}_{\text{i}}}}\right)t}\right)$$

In Equation 2, we can assume that τ_hyd_^ATP^ = τ_syn_^ATP^ because of isoenergetic ATP/ADP + P_i_ states (68). From this restriction condition, we obtained the values of time constants as τ_hyd_^ATP^ = τ_syn_^ATP^ = 0.7 ms and τ_off_^Pi^ = 1.8 ms. Furthermore, by comparing τ_syn_^ATP^ (0.7 ms) and τ_off_^Pi^ (1.8 ms), the probability of reverse reaction and the expected number of reverse reactions were estimated as 72% and 2.6 per single main pause, respectively.

In this study, we employed Scheme 1 rather than Scheme 2, because multisite catalysis occurs during unidirectional rotation of EhV_1_, and the multiple rounds of ATP cleavage/synthesis were also not considered in the previous single-molecule studies of F_1_ and V_1_ (19–29, 31–35).

## Author contributions

T. I., Y. M., H. U., and R. I. designed the experiments. T. I. performed experiments and data analysis. T. M., H. U., and F. K. contributed to sample preparation. R. I. supervised and coordinated the project and wrote the manuscript with T. I.

## Supplementary Material

Supporting Information

## Footnotes

3 The abbreviations used are:

V-ATPase: Vacuolar ATPase
ATPγS: adenosine 5′-(γ-thiotriphosphate)
EhV-ATPase: *Enterococcus hirae* V-ATPase
V_1_: V_1_-ATPase
F_1_: F_1_-ATPase
EhV_1_: *E. hirae* V_1_
TtV_1_: *T. thermophilus* V_1_
TF_1_: thermophilic *Bacillus* PS3 F_1_
HF_1_: human mitochondrial F_1_
EF_1_: *E. coli* F_1_
YF_1_: yeast mitochondrial F_1_
BF_1_: bovine mitochondrial F_1_
*K_m_*^ATP^: Michaelis constant of ATP-driven rotation
*V*_max_^ATP^: maximum velocity of ATP-driven rotation
*k*_on_^ATP^: ATP-binding rate constant
3D: three-dimensional
fps: frames per second
rps: revolutions per second
TEV: tobacco etch virus
PfGK: *P. furiosus* glucokinase
Ni-NTA: nickel-nitrilotriacetic acid.

## References

[1] Forgac M. (2007) Vacuolar ATPases: rotary proton pumps in physiology and pathophysiology. Nat. Rev. Mol. Cell Biol. 8, 917–929 10.1038/nrm2272 17912264

[2] Nishi T., and Forgac M. (2002) The vacuolar (H^+^)-ATPases—nature's most versatile proton pumps. Nat. Rev. Mol. Cell Biol. 3, 94–103 10.1038/nrm729 11836511

[3] Marshansky V., and Futai M. (2008) The V-type H^+^-ATPase in vesicular trafficking: targeting, regulation and function. Curr. Opin. Cell Biol. 20, 415–426 10.1016/j.ceb.2008.03.015 18511251 PMC7111286

[4] Cotter K., Stransky L., McGuire C., and Forgac M. (2015) Recent insights into the structure, regulation, and function of the V-ATPases. Trends Biochem. Sci. 40, 611–622 10.1016/j.tibs.2015.08.005 26410601 PMC4589219

[5] Murata T., Igarashi K., Kakinuma Y., and Yamato I. (2000) Na^+^ binding of V-type Na^+^-ATPase in *Enterococcus hirae*. J. Biol. Chem. 275, 13415–13419 10.1074/jbc.275.18.13415 10788452

[6] Yokoyama K., Muneyuki E., Amano T., Mizutani S., Yoshida M., Ishida M., and Ohkuma S. (1998) V-ATPase of *Thermus thermophilus* is inactivated during ATP hydrolysis but can synthesize ATP. J. Biol. Chem. 273, 20504–20510 10.1074/jbc.273.32.20504 9685406

[7] Grüber G., Manimekalai M. S., Mayer F., and Müller V. (2014) ATP synthases from archaea: the beauty of a molecular motor. Biochim. Biophys. Acta 1837, 940–952 10.1016/j.bbabio.2014.03.004 24650628

[8] Stewart A. G., Laming E. M., Sobti M., and Stock D. (2014) Rotary ATPases—dynamic molecular machines. Curr. Opin. Struct. Biol. 25, 40–48 10.1016/j.sbi.2013.11.013 24878343

[9] Marshansky V., Rubinstein J. L., and Grüber G. (2014) Eukaryotic V-ATPase: novel structural findings and functional insights. Biochim. Biophys. Acta 1837, 857–879 10.1016/j.bbabio.2014.01.018 24508215

[10] Zhao J., Benlekbir S., and Rubinstein J. L. (2015) Electron cryomicroscopy observation of rotational states in a eukaryotic V-ATPase. Nature 521, 241–245 10.1038/nature14365 25971514

[11] Schep D. G., Zhao J., and Rubinstein J. L. (2016) Models for the a subunits of the *Thermus thermophilus* V/A-ATPase and *Saccharomyces cerevisiae* V-ATPase enzymes by cryo-EM and evolutionary covariance. Proc. Natl. Acad. Sci. U.S.A. 113, 3245–3250 10.1073/pnas.1521990113 26951669 PMC4812769

[12] Nakanishi A., Kishikawa J. I., Tamakoshi M., Mitsuoka K., and Yokoyama K. (2018) Cryo EM structure of intact rotary H^+^-ATPase/synthase from *Thermus thermophilus*. Nat. Commun. 9, 89 10.1038/s41467-017-02553-6 29311594 PMC5758568

[13] Tsunoda J., Song C., Imai F. L., Takagi J., Ueno H., Murata T., Iino R., and Murata K. (2018) Off-axis rotor in *Enterococcus hirae* V-ATPase visualized by Zernike phase plate single-particle cryo-electron microscopy. Sci. Rep. 8, 15632 10.1038/s41598-018-33977-9 30353110 PMC6199243

[14] Murata T., Yamato I., Kakinuma Y., Leslie A. G., and Walker J. E. (2005) Structure of the rotor of the V-Type Na^+^-ATPase from *Enterococcus hirae*. Science 308, 654–659 10.1126/science.1110064 15802565

[15] Yokoyama K., and Imamura H. (2005) Rotation, structure, and classification of prokaryotic V-ATPase. J. Bioenerg. Biomembr. 37, 405–410 10.1007/s10863-005-9480-1 16691473

[16] Arai S., Saijo S., Suzuki K., Mizutani K., Kakinuma Y., Ishizuka-Katsura Y., Ohsawa N., Terada T., Shirouzu M., Yokoyama S., Iwata S., Yamato I., and Murata T. (2013) Rotation mechanism of *Enterococcus hirae* V1-ATPase based on asymmetric crystal structures. Nature 493, 703–707 10.1038/nature11778 23334411

[17] Suzuki K., Mizutani K., Maruyama S., Shimono K., Imai F. L., Muneyuki E., Kakinuma Y., Ishizuka-Katsura Y., Shirouzu M., Yokoyama S., Yamato I., and Murata T. (2016) Crystal structures of the ATP-binding and ADP-release dwells of the V1 rotary motor. Nat. Commun. 7, 13235 10.1038/ncomms13235 27807367 PMC5095293

[18] Maruyama S., Suzuki K., Imamura M., Sasaki H., Matsunami H., Mizutani K., Saito Y., Imai F. L., Ishizuka-Katsura Y., Kimura-Someya T., Shirouzu M., Uchihashi T., Ando T., Yamato I., and Murata T. (2019) Metastable asymmetrical structure of a shaftless V1 motor. Sci. Adv. 5, eaau8149 10.1126/sciadv.aau8149 30729160 PMC6353620

[19] Imamura H., Nakano M., Noji H., Muneyuki E., Ohkuma S., Yoshida M., and Yokoyama K. (2003) Evidence for rotation of V1-ATPase. Proc. Natl. Acad. Sci. U.S.A. 100, 2312–2315 10.1073/pnas.0436796100 12598655 PMC151337

[20] Imamura H., Takeda M., Funamoto S., Shimabukuro K., Yoshida M., and Yokoyama K. (2005) Rotation scheme of V1-motor is different from that of F1-motor. Proc. Natl. Acad. Sci. U.S.A. 102, 17929–17933 10.1073/pnas.0507764102 16330761 PMC1306795

[21] Minagawa Y., Ueno H., Hara M., Ishizuka-Katsura Y., Ohsawa N., Terada T., Shirouzu M., Yokoyama S., Yamato I., Muneyuki E., Noji H., Murata T., and Iino R. (2013) Basic properties of rotary dynamics of the molecular motor *Enterococcus hirae* V1-ATPase. J. Biol. Chem. 288, 32700–32707 10.1074/jbc.M113.506329 24089518 PMC3820904

[22] Yasuda R., Noji H., Yoshida M., Kinosita K. Jr, and Itoh H. (2001) Resolution of distinct rotational substeps by submillisecond kinetic analysis of F1-ATPase. Nature 410, 898–904 10.1038/35073513 11309608

[23] Shimabukuro K., Yasuda R., Muneyuki E., Hara K. Y., Kinosita K. Jr., and Yoshida M. (2003) Catalysis and rotation of F1 motor: cleavage of ATP at the catalytic site occurs in 1 ms before 40 degree substep rotation. Proc. Natl. Acad. Sci. U.S.A. 100, 14731–14736 10.1073/pnas.2434983100 14657340 PMC299784

[24] Adachi K., Oiwa K., Nishizaka T., Furuike S., Noji H., Itoh H., Yoshida M., and Kinosita K. Jr. (2007) Coupling of rotation and catalysis in F_1_-ATPase revealed by single-molecule imaging and manipulation. Cell 130, 309–321 10.1016/j.cell.2007.05.020 17662945

[25] Nishizaka T., Oiwa K., Noji H., Kimura S., Muneyuki E., Yoshida M., and Kinosita K. Jr. (2004) Chemomechanical coupling in F1-ATPase revealed by simultaneous observation of nucleotide kinetics and rotation. Nat. Struct Mol. Biol. 11, 142–148 10.1038/nsmb721 14730353

[26] Masaike T., Koyama-Horibe F., Oiwa K., Yoshida M., and Nishizaka T. (2008) Cooperative three-step motions in catalytic subunits of F_1_-ATPase correlate with 80 degrees and 40 degrees substep rotations. Nat. Struct. Mol. Biol. 15, 1326–1333 10.1038/nsmb.1510 19011636

[27] Watanabe R., Iino R., and Noji H. (2010) Phosphate release in F1-ATPase catalytic cycle follows ADP release. Nat. Chem. Biol. 6, 814–820 10.1038/nchembio.443 20871600

[28] Watanabe R., and Noji H. (2014) Timing of inorganic phosphate release modulates the catalytic activity of ATP-driven rotary motor protein. Nat. Commun. 5, 3486 10.1038/ncomms4486 24686317 PMC3988807

[29] Suzuki T., Tanaka K., Wakabayashi C., Saita E., and Yoshida M. (2014) Chemomechanical coupling of human mitochondrial F1-ATPase motor. Nat. Chem. Biol. 10, 930–936 10.1038/nchembio.1635 25242551

[30] Li C. B., Ueno H., Watanabe R., Noji H., and Komatsuzaki T. (2015) ATP hydrolysis assists phosphate release and promotes reaction ordering in F1-ATPase. Nat. Commun. 6, 10223 10.1038/ncomms10223 26678797 PMC4703894

[31] Noji H., Häsler K., Junge W., Kinosita K. Jr., Yoshida M., and Engelbrecht S. (1999) Rotation of *Escherichia coli* F_1_-ATPase. Biochem. Biophys. Res. Commun. 260, 597–599 10.1006/bbrc.1999.0885 10403811

[32] Nakanishi-Matsui M., Kashiwagi S., Ubukata T., Iwamoto-Kihara A., Wada Y., and Futai M. (2007) Rotational catalysis of *Escherichia coli* ATP synthase F1 sector: stochastic fluctuation and a key domain of the β subunit. J. Biol. Chem. 282, 20698–20704 10.1074/jbc.M700551200 17517893

[33] Bilyard T., Nakanishi-Matsui M., Steel B. C., Pilizota T., Nord A. L., Hosokawa H., Futai M., and Berry R. M. (2013) High-resolution single-molecule characterization of the enzymatic states in *Escherichia coli* F1-ATPase. Philos. Trans. R. Soc. Lond. B Biol. Sci. 368, 20120023 10.1098/rstb.2012.0023 23267177 PMC3538426

[34] Steel B. C., Nord A. L., Wang Y., Pagadala V., Mueller D. M., and Berry R. M. (2015) Comparison between single-molecule and X-ray crystallography data on yeast F1-ATPase. Sci. Rep. 5, 8773 10.1038/srep08773 25753753 PMC4894397

[35] Martin J. L., Ishmukhametov R., Hornung T., Ahmad Z., and Frasch W. D. (2014) Anatomy of F1-ATPase powered rotation. Proc. Natl. Acad. Sci. U.S.A. 111, 3715–3720 10.1073/pnas.1317784111 24567403 PMC3956197

[36] Abrahams J. P., Leslie A. G., Lutter R., and Walker J. E. (1994) Structure at 2.8 Å resolution of F1-ATPase from bovine heart mitochondria. Nature 370, 621–628 10.1038/370621a0 8065448

[37] Menz R. I., Walker J. E., and Leslie A. G. (2001) Structure of bovine mitochondrial F_1_-ATPase with nucleotide bound to all three catalytic sites: implications for the mechanism of rotary catalysis. Cell 106, 331–341 10.1016/S0092-8674(01)00452-4 11509182

[38] Kagawa R., Montgomery M. G., Braig K., Leslie A. G., and Walker J. E. (2004) The structure of bovine F1-ATPase inhibited by ADP and beryllium fluoride. EMBO J. 23, 2734–2744 10.1038/sj.emboj.7600293 15229653 PMC514953

[39] Bowler M. W., Montgomery M. G., Leslie A. G., and Walker J. E. (2007) Ground state structure of F1-ATPase from bovine heart mitochondria at 1.9 Å resolution. J. Biol. Chem. 282, 14238–14242 10.1074/jbc.M700203200 17350959

[40] Rees D. M., Montgomery M. G., Leslie A. G., and Walker J. E. (2012) Structural evidence of a new catalytic intermediate in the pathway of ATP hydrolysis by F1-ATPase from bovine heart mitochondria. Proc. Natl. Acad. Sci. U.S.A. 109, 11139–11143 10.1073/pnas.1207587109 22733764 PMC3396519

[41] Bason J. V., Montgomery M. G., Leslie A. G., and Walker J. E. (2015) How release of phosphate from mammalian F1-ATPase generates a rotary substep. Proc. Natl. Acad. Sci. U.S.A. 112, 6009–6014 10.1073/pnas.1506465112 25918412 PMC4434703

[42] Zhou A., Rohou A., Schep D. G., Bason J. V., Montgomery M. G., Walker J. E., Grigorieff N., and Rubinstein J. L. (2015) Structure and conformational states of the bovine mitochondrial ATP synthase by cryo-EM. Elife 4, e10180 10.7554/eLife.10180 26439008 PMC4718723

[43] Kabaleeswaran V., Puri N., Walker J. E., Leslie A. G., and Mueller D. M. (2006) Novel features of the rotary catalytic mechanism revealed in the structure of yeast F1 ATPase. EMBO J. 25, 5433–5442 10.1038/sj.emboj.7601410 17082766 PMC1636620

[44] Kabaleeswaran V., Shen H., Symersky J., Walker J. E., Leslie A. G., and Mueller D. M. (2009) Asymmetric structure of the yeast F1 ATPase in the absence of bound nucleotides. J. Biol. Chem. 284, 10546–10551 10.1074/jbc.M900544200 19233840 PMC2667741

[45] Arsenieva D., Symersky J., Wang Y., Pagadala V., and Mueller D. M. (2010) Crystal structures of mutant forms of the yeast F1 ATPase reveal two modes of uncoupling. J. Biol. Chem. 285, 36561–36569 10.1074/jbc.M110.174383 20843806 PMC2978584

[46] Robinson G. C., Bason J. V., Montgomery M. G., Fearnley I. M., Mueller D. M., Leslie A. G., and Walker J. E. (2013) The structure of F_1_-ATPase from *Saccharomyces cerevisiae* inhibited by its regulatory protein IF_1_. Open Biol. 3, 120164 10.1098/rsob.120164 23407639 PMC3603450

[47] Cingolani G., and Duncan T. M. (2011) Structure of the ATP synthase catalytic complex (F_1_) from *Escherichia coli* in an autoinhibited conformation. Nat. Struct. Mol. Biol. 18, 701–707 10.1038/nsmb.2058 21602818 PMC3109198

[48] Sobti M., Smits C., Wong A. S., Ishmukhametov R., Stock D., Sandin S., and Stewart A. G. (2016) Cryo-EM structures of the autoinhibited *E. coli* ATP synthase in three rotational states. Elife 5, e21598 10.7554/eLife.21598 28001127 PMC5214741

[49] Shirakihara Y., Shiratori A., Tanikawa H., Nakasako M., Yoshida M., and Suzuki T. (2015) Structure of a thermophilic F1-ATPase inhibited by an ϵ-subunit: deeper insight into the ϵ-inhibition mechanism. FEBS J. 282, 2895–2913 10.1111/febs.13329 26032434

[50] Guo H., Suzuki T., and Rubinstein J. L. (2019) Structure of a bacterial ATP synthase. Elife 8, e43128 10.7554/eLife.43128 30724163 PMC6377231

[51] Furuike S., Nakano M., Adachi K., Noji H., Kinosita K. Jr., and Yokoyama K. (2011) Resolving stepping rotation in *Thermus thermophilus* H^+^-ATPase/synthase with an essentially drag-free probe. Nat. Commun. 2, 233 10.1038/ncomms1215 21407199 PMC3072102

[52] Numoto N., Hasegawa Y., Takeda K., and Miki K. (2009) Inter-subunit interaction and quaternary rearrangement defined by the central stalk of prokaryotic V1-ATPase. EMBO Rep. 10, 1228–1234 10.1038/embor.2009.202 19779483 PMC2775172

[53] Nagamatsu Y., Takeda K., Kuranaga T., Numoto N., and Miki K. (2013) Origin of asymmetry at the intersubunit interfaces of V1-ATPase from *Thermus thermophilus*. J. Mol. Biol. 425, 2699–2708 10.1016/j.jmb.2013.04.022 23639357

[54] Zhou L., and Sazanov L. A. (2019) Structure and conformational plasticity of the intact *Thermus thermophilus* V/A-type ATPase. Science 365, eaaw9144 10.1126/science.aaw9144 31439765

[55] Ueno H., Minagawa Y., Hara M., Rahman S., Yamato I., Muneyuki E., Noji H., Murata T., and Iino R. (2014) Torque generation of *Enterococcus hirae* V-ATPase. J. Biol. Chem. 289, 31212–31223 10.1074/jbc.M114.598177 25258315 PMC4223323

[56] Iino R., Ueno H., Minagawa Y., Suzuki K., and Murata T. (2015) Rotational mechanism of *Enterococcus hirae* V1-ATPase by crystal-structure and single-molecule analyses. Curr. Opin. Struct. Biol. 31, 49–56 10.1016/j.sbi.2015.02.013 25796033

[57] Beckett D., Kovaleva E., and Schatz P. J. (1999) A minimal peptide substrate in biotin holoenzyme synthetase-catalyzed biotinylation. Protein Sci. 8, 921–929 10.1110/ps.8.4.921 10211839 PMC2144313

[58] Komoriya Y., Ariga T., Iino R., Imamura H., Okuno D., and Noji H. (2012) Principal role of the arginine finger in rotary catalysis of F1-ATPase. J. Biol. Chem. 287, 15134–15142 10.1074/jbc.M111.328153 22403407 PMC3340237

[59] Iino R., and Noji H. (2013) Intersubunit coordination and cooperativity in ring-shaped NTPases. Curr. Opin. Struct. Biol. 23, 229–234 10.1016/j.sbi.2013.01.004 23395511

[60] Yukawa A., Iino R., Watanabe R., Hayashi S., and Noji H. (2015) Key chemical factors of arginine finger catalysis of F1-ATPase clarified by an unnatural amino acid mutation. Biochemistry 54, 472–480 10.1021/bi501138b 25531508

[61] Seong I. S., Oh J. Y., Yoo S. J., Seol J. H., and Chung C. H. (1999) ATP-dependent degradation of SulA, a cell division inhibitor, by the HslVU protease in *Escherichia coli*. FEBS Lett. 456, 211–214 10.1016/S0014-5793(99)00935-7 10452560

[62] Burton R. E., Baker T. A., and Sauer R. T. (2003) Energy-dependent degradation: Linkage between ClpX-catalyzed nucleotide hydrolysis and protein-substrate processing. Protein Sci. 12, 893–902 10.1110/ps.0237603 12717012 PMC2323860

[63] Kon T., Mogami T., Ohkura R., Nishiura M., and Sutoh K. (2005) ATP hydrolysis cycle-dependent tail motions in cytoplasmic dynein. Nat. Struct. Mol. Biol. 12, 513–519 10.1038/nsmb930 15880123

[64] Liu C. W., Li X., Thompson D., Wooding K., Chang T. L., Tang Z., Yu H., Thomas P. J., and DeMartino G. N. (2006) ATP binding and ATP hydrolysis play distinct roles in the function of 26S proteasome. Mol. Cell 24, 39–50 10.1016/j.molcel.2006.08.025 17018291 PMC3951175

[65] Gates S. N., Yokom A. L., Lin J., Jackrel M. E., Rizo A. N., Kendsersky N. M., Buell C. E., Sweeny E. A., Mack K. L., Chuang E., Torrente M. P., Su M., Shorter J., and Southworth D. R. (2017) Ratchet-like polypeptide translocation mechanism of the AAA+ disaggregase Hsp104. Science 357, 273–279 10.1126/science.aan1052 28619716 PMC5770238

[66] Uchihashi T., Watanabe Y. H., Nakazaki Y., Yamasaki T., Watanabe H., Maruno T., Ishii K., Uchiyama S., Song C., Murata K., Iino R., and Ando T. (2018) Dynamic structural states of ClpB involved in its disaggregation function. Nat. Commun. 9, 2147 10.1038/s41467-018-04587-w 29858573 PMC5984625

[67] Shimabukuro K., Muneyuki E., and Yoshida M. (2006) An alternative reaction pathway of F1-ATPase suggested by rotation without 80 degrees/40 degrees substeps of a sluggish mutant at low ATP. Biophys. J. 90, 1028–1032 10.1529/biophysj.105.067298 16258036 PMC1367089

[68] Boyer P. D., Cross R. L., and Momsen W. (1973) A new concept for energy coupling in oxidative phosphorylation based on a molecular explanation of the oxygen exchange reactions. Proc. Natl. Acad. Sci. U.S.A. 70, 2837–2839 10.1073/pnas.70.10.2837 4517936 PMC427120

[69] Choate G. L., Hutton R. L., and Boyer P. D. (1979) Occurrence and significance of oxygen exchange reactions catalyzed by mitochondrial adenosine triphosphatase preparations. J. Biol. Chem. 254, 286–290 153910

[70] Boyer P. D. (1987) The unusual enzymology of ATP synthase. Biochemistry 26, 8503–8507 10.1021/bi00400a001 2894841

[71] Gresser M. J., Myers J. A., and Boyer P. D. (1982) Catalytic site cooperativity of beef heart mitochondrial F1 adenosine triphosphatase: correlations of initial velocity, bound intermediate, and oxygen exchange measurements with an alternating three-site model. J. Biol. Chem. 257, 12030–12038 6214554

[72] Rondelez Y., Tresset G., Nakashima T., Kato-Yamada Y., Fujita H., Takeuchi S., and Noji H. (2005) Highly coupled ATP synthesis by F1-ATPase single molecules. Nature 433, 773–777 10.1038/nature03277 15716957

[73] Sielaff H., Martin J., Singh D., Biuković G., Grüber G., and Frasch W. D. (2016) Power stroke angular velocity profiles of archaeal A-ATP synthase *versus* thermophilic and mesophilic F-ATP synthase molecular motors. J. Biol. Chem. 291, 25351–25363 10.1074/jbc.M116.745240 27729450 PMC5207238

[74] Kengen S. W., de Bok F. A., van Loo N. D., Dijkema C., Stams A. J., and de Vos W. M. (1994) Evidence for the operation of a novel Embden-Meyerhof pathway that involves ADP-dependent kinases during sugar fermentation by *Pyrococcus furiosus*. J. Biol. Chem. 269, 17537–17541 8021261

[75] Kengen S. W., Tuininga J. E., de Bok F. A., Stams A. J., and de Vos W. M. (1995) Purification and characterization of a novel ADP-dependent glucokinase from the hyperthermophilic archaeon *Pyrococcus furiosus*. J. Biol. Chem. 270, 30453–30457 10.1074/jbc.270.51.30453 8530474

[76] Ito S., Fushinobu S., Yoshioka I., Koga S., Matsuzawa H., and Wakagi T. (2001) Structural basis for the ADP-specificity of a novel glucokinase from a hyperthermophilic archaeon. Structure 9, 205–214 10.1016/S0969-2126(01)00577-9 11286887

[77] Ito S., Fushinobu S., Jeong J. J., Yoshioka I., Koga S., Shoun H., and Wakagi T. (2003) Crystal structure of an ADP-dependent glucokinase from *Pyrococcus furiosus*: implications for a sugar-induced conformational change in ADP-dependent kinase. J. Mol. Biol. 331, 871–883 10.1016/S0022-2836(03)00792-7 12909015

[78] Ueno H., Nishikawa S., Iino R., Tabata K. V., Sakakihara S., Yanagida T., and Noji H. (2010) Simple dark-field microscopy with nanometer spatial precision and microsecond temporal resolution. Biophys. J. 98, 2014–2023 10.1016/j.bpj.2010.01.011 20441766 PMC2862163

[79] Nakamura A., Okazaki K. I., Furuta T., Sakurai M., and Iino R. (2018) Processive chitinase is Brownian monorail operated by fast catalysis after peeling rail from crystalline chitin. Nat. Commun. 9, 3814 10.1038/s41467-018-06362-3 30232340 PMC6145945

[80] Ando J., Nakamura A., Visootsat A., Yamamoto M., Song C., Murata K., and Iino R. (2018) Single-nanoparticle tracking with angstrom localization precision and microsecond time resolution. Biophys. J. 115, 2413–2427 10.1016/j.bpj.2018.11.016 30527446 PMC6302141

