# Supplementary material for: Single-molecule analysis reveals rotational substeps and chemo-mechanical coupling scheme of Enterococcus hirae V1-ATPase
Source: J Biol Chem. 2019 Sep 13;294(45):17017–30. doi: 10.1074/jbc.RA119.008947 (PMC6851342; doi:10.1074/jbc.RA119.008947)
Supplement: Supporting Information [file supp_294_45_17017__index.html]

Single-molecule analysis reveals rotational substeps and chemo-mechanical coupling scheme of Enterococcus hirae V1-ATPase — Chemo-mechanical coupling of E. hirae V1-ATPase — Single-molecule analysis reveals rotational substeps and chemo-mechanical coupling scheme of Enterococcus hirae V1-ATPase — Chemo-mechanical coupling of E. hirae V1-ATPase — Supporting Information 

# Single-molecule analysis reveals rotational substeps and chemo-mechanical coupling scheme of *Enterococcus hirae* V1-ATPase

## Supporting Information

- Supporting Information (to be published online) - Supporting Figures S1 to S7.
