# Supplementary material for: Single-molecule analysis reveals rotational substeps and chemo-mechanical coupling scheme of Enterococcus hirae V1-ATPase
Source: J Biol Chem. 2019 Sep 13;294(45):17017–30. doi: 10.1074/jbc.RA119.008947 (PMC6851342; doi:10.1074/jbc.RA119.008947)
Supplement: Supporting Information [file supp_RA119.008947_145001_2_supp_394502_pxrg66.pdf]

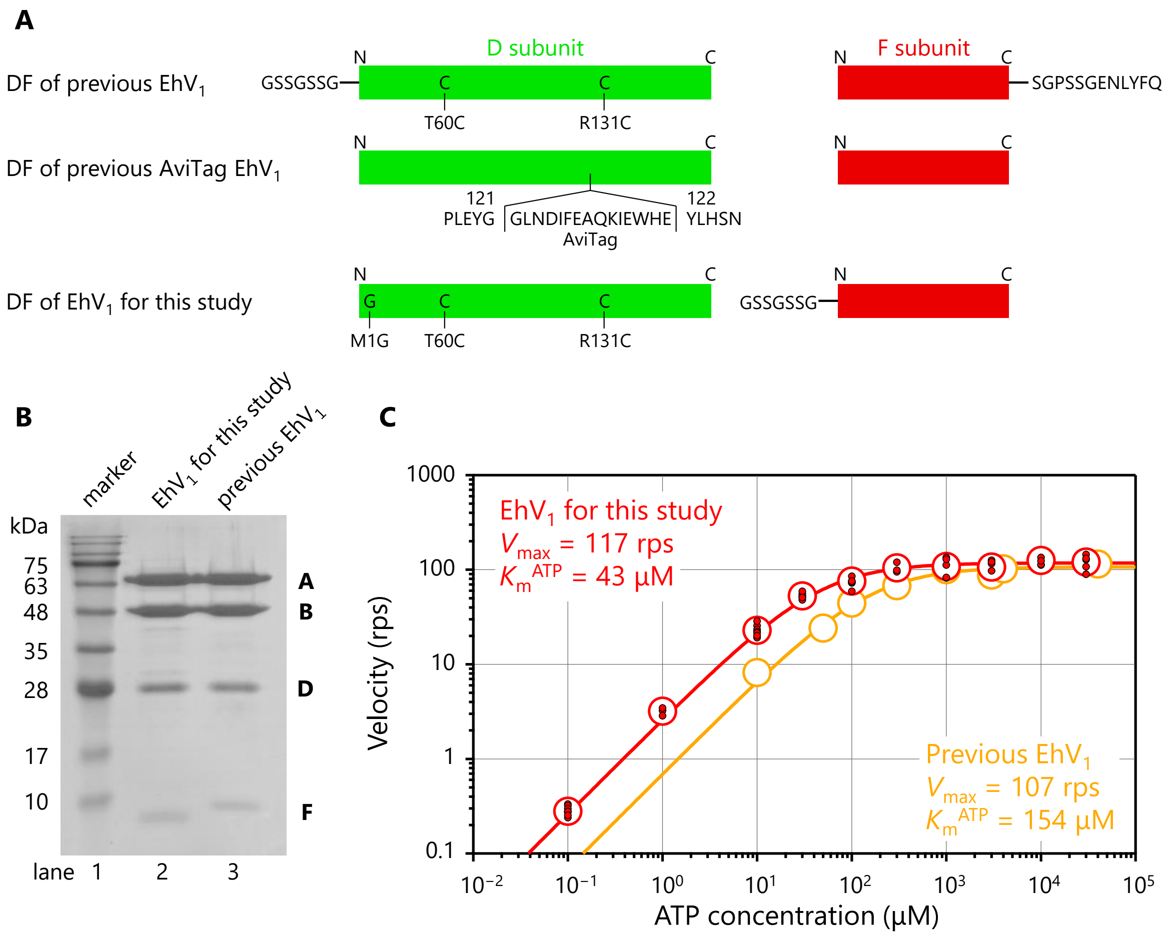

Fig. S1. Comparison between new EhV_1 construct used in this study and those used in previous study. (*A*) Differences in the construct design among the DF subcomplexes of EhV_1 and AviTag EhV_1 used in the previous study, and newly constructed EhV_1 used in this study. The previous EhV_1 construct has additional 7 amino acid residues (GSSGSSG) at the N-terminus of the D subunit and 12 amino acid residues (SGPSSGENLYFQ) at the C-terminus of the F subunit, after removal of Riken-His tags for purification. Another previous construct, AviTag EhV_1 has the AviTag which consists of 15 amino acid residues (GLNDIFEAQKIEWHE) inserted between Gly121 and Tyr122 of the D subunit for biotinylation. In the newly-constructed EhV_1, Met1 of the D subunit was mutated to glycine to eliminate additional amino acid residues at the N-terminus, and Riken-His tag for purification of the F subunit was transferred from C-terminus to N-terminus to make the additional amino acid residues short. (*B*) SDS-PAGE of reconstituted EhV_1. Lanes 1, 2, 3 correspond to marker, newly constructed EhV_1, and the previous EhV_1, respectively. The molecular masses of the A and B subunits are 65 and 51 kDa, respectively. Molecular masses of the D and F subunits are 24 and 12 kDa for new construct, and 25 and 13 kDa for the previous construct, respectively. (*C*) [ATP] dependences of rotation velocity of newly constructed EhV_1 (red, also shown in Fig. 2A) and EhV_1 used in the previous study (orange). The open circles and small filled circles show the average velocities and velocities of individual molecules, respectively. The curves indicate the fit with the Michaelis-Menten equation: $V = V_{max}^{ATP} \times [ATP] / (K_m^{ATP} + [ATP])$. The V_{max}^{ATP} and K_m^{ATP} were 117 ± 3 rps (fitted parameter ± fitting error) and 43 ± 6 μM for new construct, and 107 ± 4 rps and 154 ± 33 μM for the previous construct. The k_{on}^{ATP} was estimated from $3 \times V_{max}^{ATP} / K_m^{ATP}$ as 8.2×10^6 $M^{-1}s^{-1}$ for new construct, and 2.2×10^6 $M^{-1}s^{-1}$ for the previous construct.

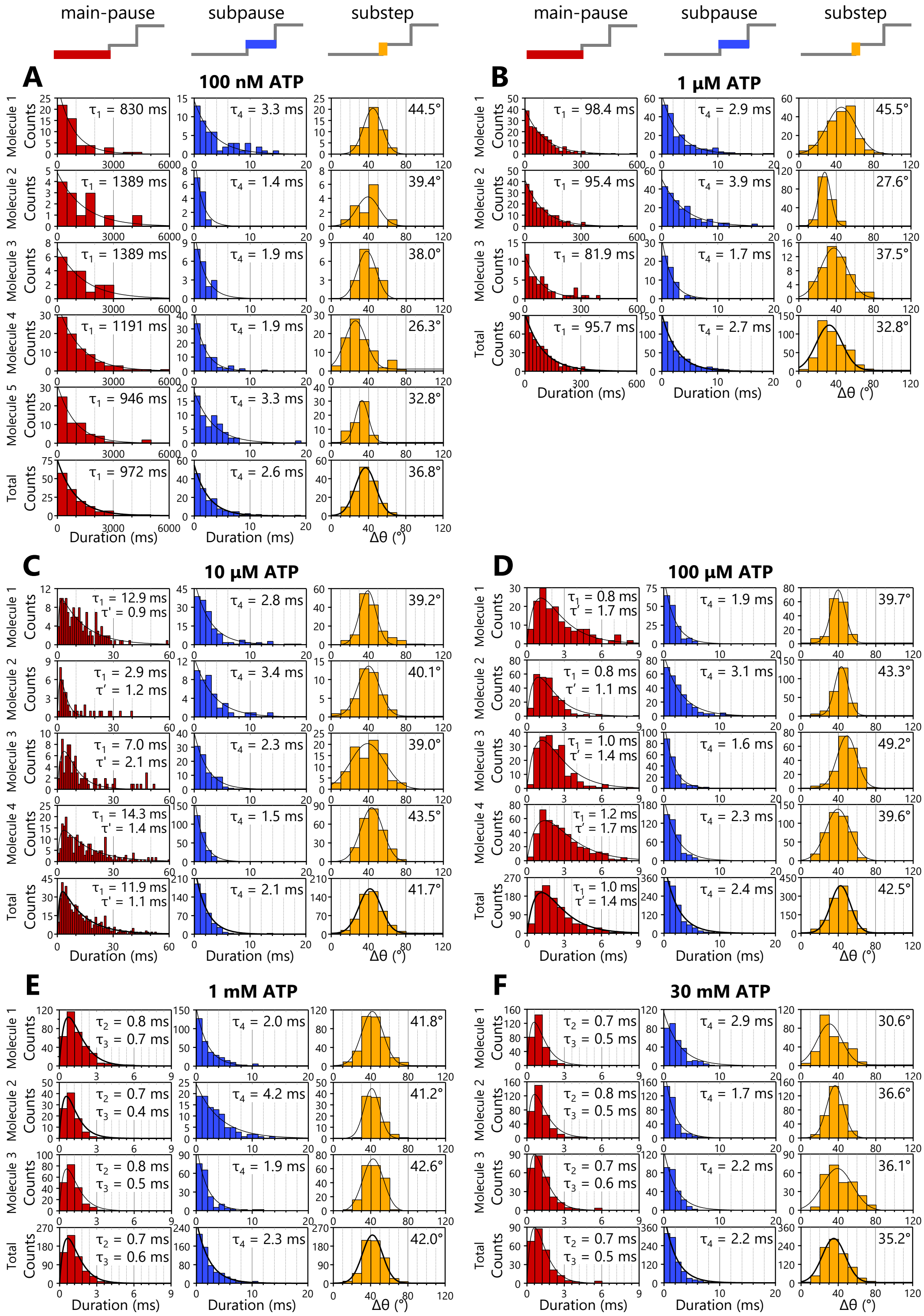

Fig. S2. Distributions of duration time for main-pause and subpause, and Δθ for individual molecules. Distributions of duration time for the main-pause (*red*), subpause (*blue*), and angle difference (Δθ) from main-pause to subpause (*orange*) at (*A*) 100 nM, (*B*) 1 μM, (*C*) 10 μM, (*D*) 100 μM, (*E*) 1 mM, and (*F*) 30 mM ATP shown in Fig. 2B-D. The distributions of duration time for the main-pause were fitted with single exponential decay functions: constant × exp(−t/τ) for (*A*) and (*B*), and with double-exponential decay functions assuming two consecutive first-order reactions: constant × (exp(−t/τ) − exp(−t/τ')) for (*C*) to (*F*). The distributions of duration time for the subpause were fitted with single exponential decay functions: constant × exp(−t/τ). The distributions of Δθ were fitted with Gaussian function. The fitted parameters were shown in each distribution as an inset.

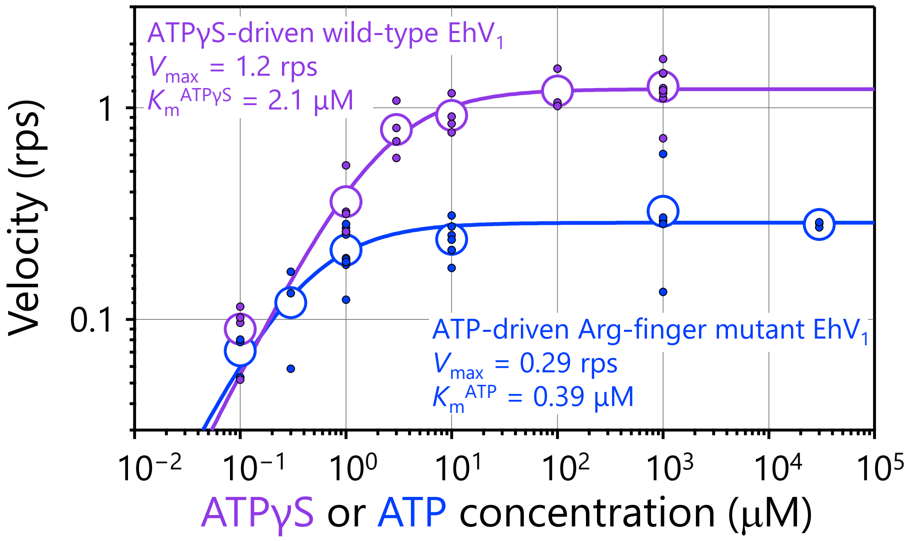

Fig. S3. Rotation velocities of wild-type EhV_1 driven by ATPγS and Arg-finger mutant driven by ATP. [ATPγS] dependence of rotation velocity of wild-type EhV_1 (purple) and [ATP] dependence of rotation velocity of Arg-finger mutant (blue) are shown. The open circles and filled small circles show the average velocities and velocities of individual molecules, respectively. The curves indicate the fit with the Michaelis-Menten equation: $V = V_{max}^{S} \times [S] / (K_{m}^{S} + [S]$, S is ATPγS or ATP). For the rotation of wild-type EhV_1 driven by ATPγS, the $V_{max}^{ATP\gamma S}$ and $K_{m}^{ATP\gamma S}$ were 1.2 ± 0.1 rps (fitted parameter ± fitting error) and 2.1 ± 0.4 μM, respectively. For the rotation of Arg-finger mutant driven by ATP, the V_{max}^{ATP} and K_{m}^{ATP} were 0.29 ± 0.02 rps and 0.38 ± 0.1 μM, respectively. The $k_{on}^{ATP\gamma S}$ for the rotation of wild-type EhV_1 driven by ATPγS and the k_{on}^{ATP} for the rotation Arg-finger mutant driven by ATP were estimated as 1.7×10^{6} $M^{-1}s^{-1}$ and 2.3×10^{6} $M^{-1}s^{-1}$, respectively from $3 \times V_{max}^{S} / K_{m}^{S}$.

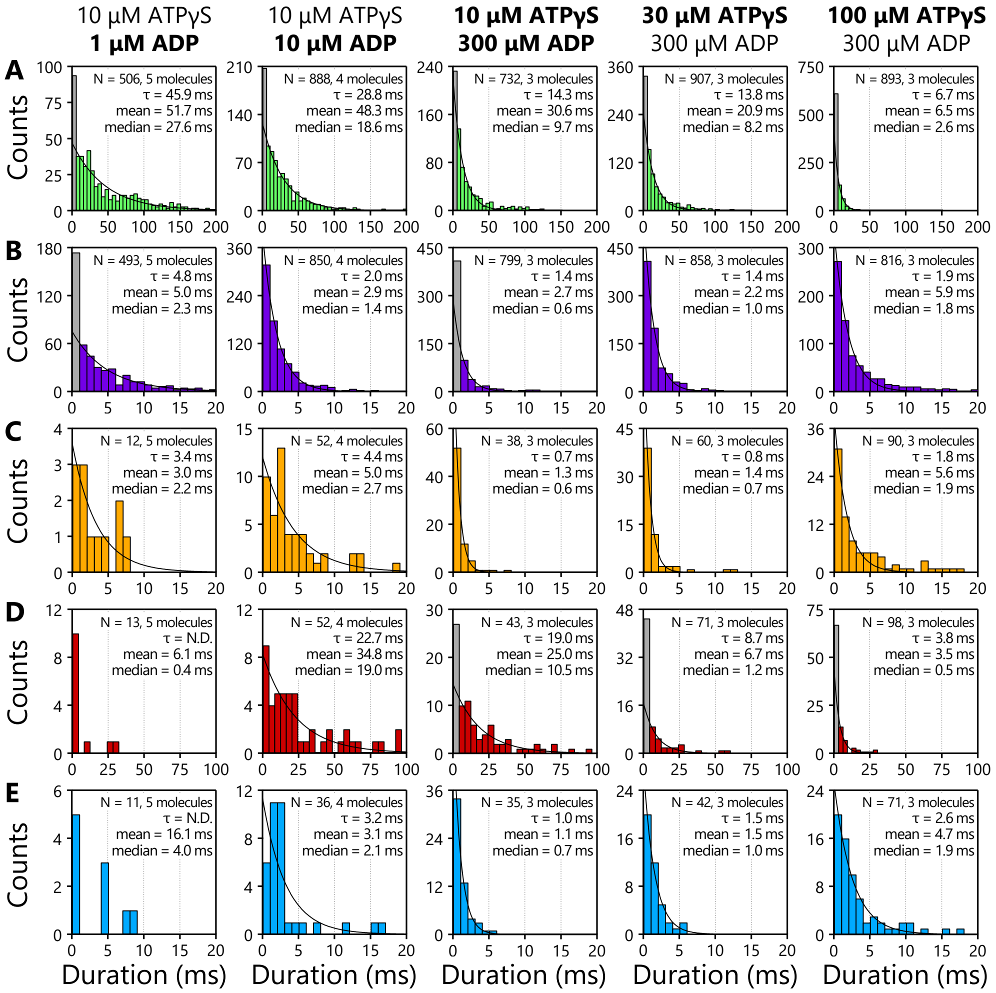

Fig. S4. Distributions of duration before and after backward steps in ATPγS -driven rotation of wild-type EhV_1 in the presence of ADP.

Distributions of duration for pauses (Fig. 4B) at 10 μM ATPγS in the presence of 1, 10, and 300 μM ADP, or 10, 30, and 100 μM ATPγS in the presence of 300 μM ADP are shown. Time constants τ estimated by fitting with single exponential decay functions, mean and median values are shown. The first bins shown in gray were not included for fitting. Some distributions were not well fitted with single exponential decay functions due to insufficient number of events, and time constants were not determined (N.D.). (*A*) Pause before −80° backward step. (*B*) Pause before +80° recovery step after −80° backward step. (*C*) Pause before −40° backward step. (*D*) Pause before +40° recovery step. (*E*) Pause before +80° recovery step after +40° recovery step.

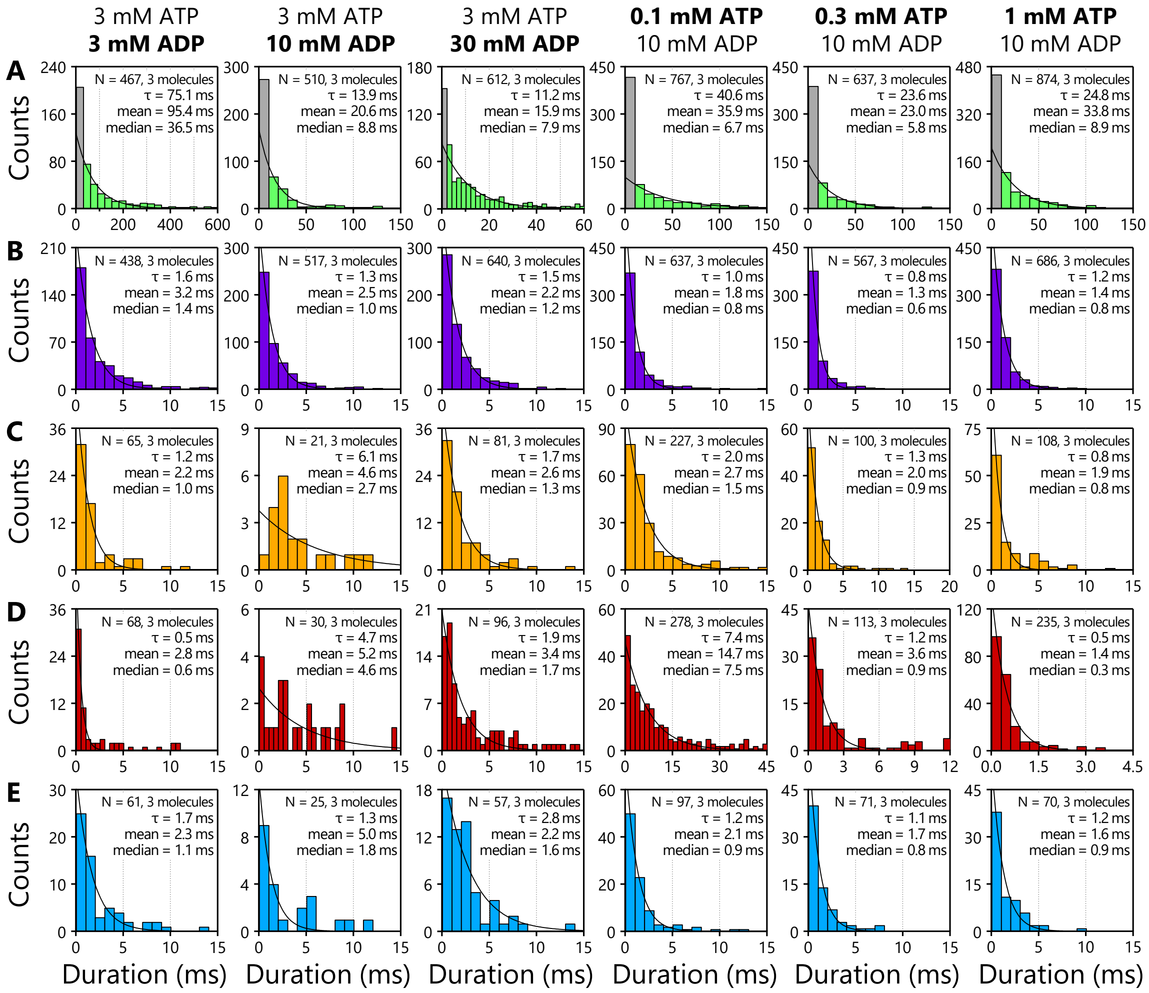

Fig. S5. Distributions of duration before and after backward step in the ATP-driven rotation of Arg-finger mutant EhV_1 in the presence of ADP.

Distributions of durations for pauses (Fig. 5B) at 3 mM ATP in the presence of 3, 10, and 30 mM ADP or 0.1, 0.3, and 1 mM ATP in the presence of 10 mM ADP. Time constants τ estimated by fitting with single exponential decay functions, mean and median values are shown. The first bins shown in gray were not included for fitting. (*A*) Pause before −80° backward step. (*B*) Pause before +80° recovery step after −80° backward step. (*C*) Pause before −40° backward step. (*D*) Pause before +40° recovery step. (*E*) Pause before +80° recovery step after +40° recovery step.

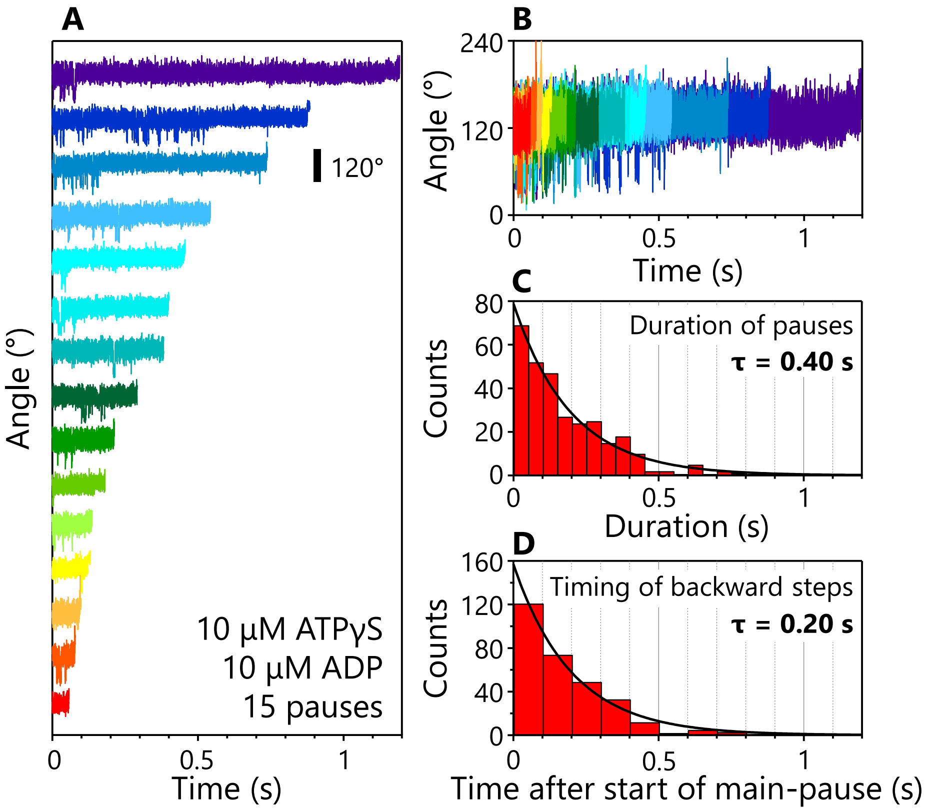

Fig. S6. Timing of the backward step during the main-pause.
(*A*) The fifteen representative time courses of the main-pause of a rotating EhV_1 at 10 μM ATPγS in the presence of 10 μM ADP. (*B*) The superimposed traces of *A*. The colors of each pause correspond to those in *A*. (*C*) Distribution of duration of the main-pause. The curve shows the fits by single exponential decay function and time constant was estimated as 0.40 ± 0.02 s (fitted parameter ± fitting error). (*D*) Distribution of timing of backward steps after start of the main-pause. The curve shows the fits by single exponential decay function and time constant estimated as 0.20 ± 0.01 s.

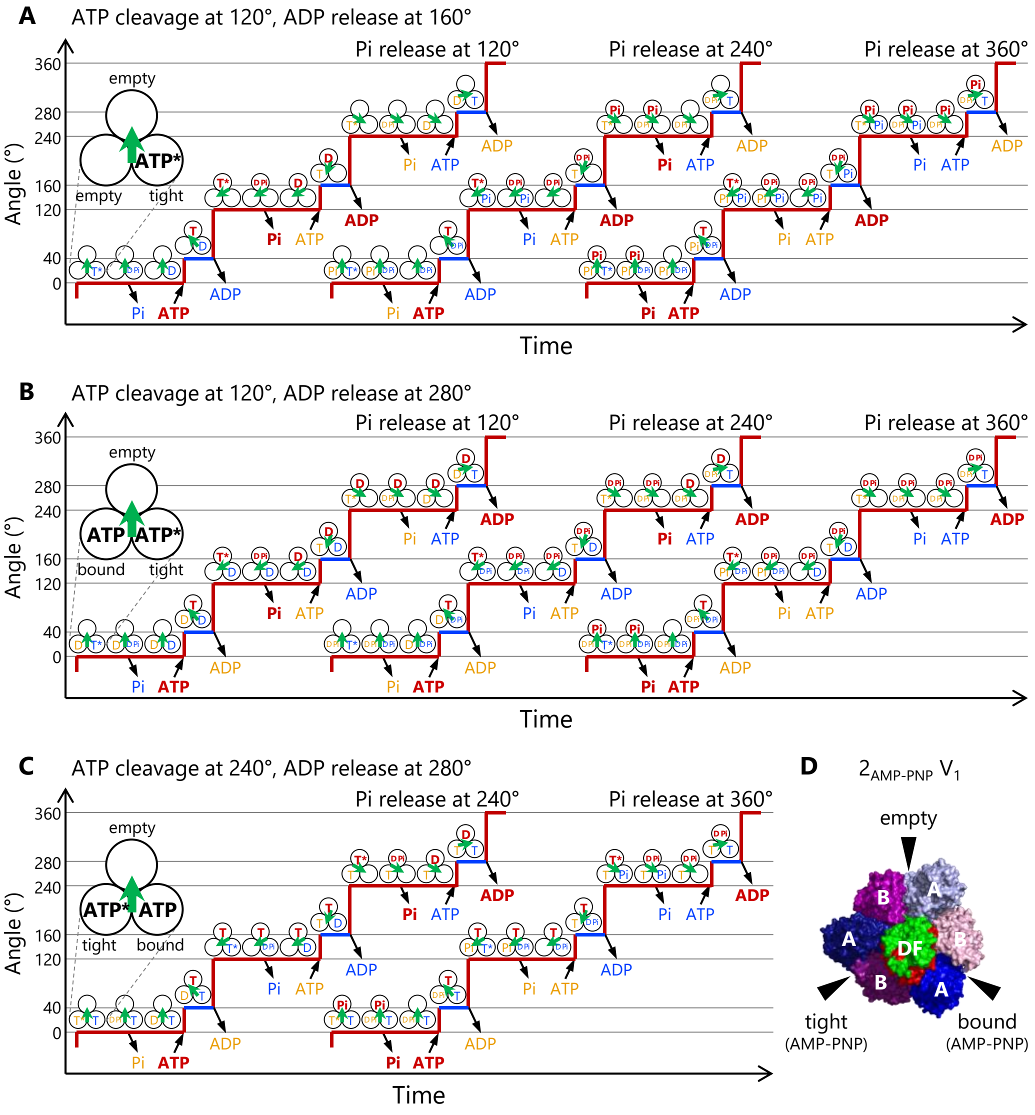

Fig. S7. Possible models of chemo-mechanical coupling scheme of EhV_1.

After ATP binding at 0°, following models are possible. (*A*) ATP is cleaved at 120° and ADP is released at 160°. (*B*) ATP is cleaved at 120° and ADP is released at 280°. For the models shown in *A* and *B*, Pi can be released at 120°, 240°, or 360°. (*C*) ATP is cleaved at 240° and ADP is released at 280°. In *C*, Pi can be released at 240° or 360°. (*D*) The crystal structure of EhV_1 which binds with two AMP-PNP molecules at the two catalytic sites among three (tight and bound sites, PDB ID: 3VR6).
